# The Critical Point and the Supercritical State of Alkali Feldspars: Implications for the Behavior of the Crust During Impacts

**DOI:** 10.1029/2020JE006412

**Published:** 2020-09-15

**Authors:** Anaïs Kobsch, Razvan Caracas

**Affiliations:** ^1^ CNRS, École Normale Supérieure de Lyon, Laboratoire de Géologie de Lyon Lyon France; ^2^ The Centre for Earth Evolution and Dynamics (CEED) University of Oslo Oslo Norway

**Keywords:** feldspars, giant impact, supercritical, phase diagram, density functional theory

## Abstract

The position of the vapor‐liquid dome and of the critical point determine the evolution of the outermost parts of the protolunar disk during cooling and condensation after the Giant Impact. The parts of the disk in supercritical or liquid state evolve as a single thermodynamic phase; when the thermal trajectory of the disk reaches the liquid‐vapor dome, gas and melt separate leading to heterogeneous convection and phase separation due to friction. Different layers of the proto‐Earth behaved differently during the Giant Impact depending on their constituent materials and initial thermodynamic conditions. Here we use first‐principles molecular dynamics to determine the position of the critical point for NaAlSi_3_O_8_ and KAlSi_3_O_8_ feldspars, major minerals of the Earth and Moon crusts. The variations of the pressure calculated at various volumes along isotherms yield the position of the critical points: 0.5–0.8 g cm^−3^ and 5500–6000 K range for the Na‐feldspar, 0.5–0.9 g cm^−3^ and 5000–5500 K range for the K‐feldspar. The simulations suggest that the vaporization is incongruent, with a degassing of O_2_ starting at 4000 K and gas component made mostly of free Na and K cations, O_2_, SiO and SiO_2_ species for densities below 1.5 g cm^−3^. The Hugoniot equations of state imply that low‐velocity impactors (<8.3 km s^−1^) would at most melt a cold feldspathic crust, whereas large impacts in molten crust would see temperatures raise up to 30000 K.

## Key Points

- First‐principles molecular dynamics simulations of feldspars at low density and high temperature
- Free Na, K, and O_2_, SiO, or SiO_2_ constitute the first gas when the vaporization starts
- The critical point of alkali feldspars is between 0.5 and 0.9 g cm^−3^ and 5000–6000 K

## 1 Introduction

For more than 20 years, the simulations of formation of the Moon from an impact‐generated disk have made huge progress and went through many different models, from the canonical impact (Canup & Esposito, 1996) to the high‐energy high‐angular‐momentum impact (Canup, 2012; Ćuk & Stewart, 2012) and more recently to the formation of a synestia (Lock et al., 2018). They tend to reproduce better and better the observed features of the actual Earth‐Moon system, like the angular momentum or the fractionation of some isotopes and elements. Even if each simulation uses different parameters, they all model the mantle and the core using respectively particles of dunite or forsterite and of iron, with the equations of state provided by the M‐ANEOS package (Melosh, 2007).

For many years shock experiments improved equations of state, which are major parameters of the hydrodynamics simulations, on a variety of major geological materials, like MgSiO_3_ glass, enstatite and olivine (Luo et al., 2004), silica (Kraus et al., 2012), and MgO (Root et al., 2015). These experiments sample points along the shock Hugoniot equations of state, at high temperatures and pressures, typical for the peak conditions attained during the shock. In the laboratory their timescales are on the order of nanoseconds, which makes many measurements hard to realize. Moreover, they can rarely follow the evolution of the sample after the shock release, when the pressure and temperature decrease along quasi‐isentropic lines over time scales on the order of minutes. Combined with long sample and apparatus preparation times and few available shock facilities, such experiments are relatively scarce. Here we take a numerical approach and employ first‐principles molecular dynamics (FPMD) simulations. We study the behavior of two feldspar end‐members terms in this regime.

Feldspars are aluminosilicates with general formula (Ca,Na,K) (Al,Si)_4_O_8_. Based on the major‐element composition of the current terrestrial bulk continental crust, its normative mineralogy would consist of 58.9% feldspars by weight. The Moon highland crust is made of at least 80% of ferroan anorthosite, a rock composed almost entirely of Ca‐end‐member plagioclase (Taylor & McLennan, 2008). On Venus spectroscopic studies show the presence of a mixture of phases that includes albite. This should be a relatively stable phase compared to Ca‐bearing minerals, like anorthite, which would easily react with the atmosphere (Gilmore et al., 2017). Surface models of Mercury show the presence of up to 70% of Na‐rich plagioclase feldspar (Warell et al., 2010). Feldspar minerals are also widely present in eucrites, on Vesta, etc. (e.g., Szurgot, 2014). Thus, feldspars represent major mineral components of the crust of terrestrial bodies.

Because of their importance, feldspars have been widely studied for more than a century. The first melting experiments were done in the beginning of the last century on plagioclase feldspars (Bowen, 1913), in which Ca‐end‐members and Na‐end‐members melted at about 1823 and 1373 K, respectively. Ever since, a plethora of experiments (e.g., Akaogi et al., 2004; Bell & Roseboom, 1969; Lindsley, 1966; Newton & Smith, 1967; Urakawa et al., 1994) determined the melting curves of the entire series, investigated the behavior of the melt in various assemblages, and analyzed the compressibility of feldspathic glasses up to at least 128 GPa.

The Hugoniot equations of state for the three end‐members and several intermediate compositions were investigated through shock experiments (e.g., Ahrens et al., 1969; Asimow & Ahrens, 2010; McQueen et al., 1967). The maximum shock conditions reached in these experiments were about 120 GPa and 6000 K.

More recently, molecular dynamics (MD) simulations gave access to a series of thermodynamic properties, including the structure and the dynamics of the melts over a large range of temperatures (2500–6100 K) and pressures (0–160 GPa), using either classical MD (e.g., Neilson et al., 2016; Spera et al., 2009) or FPMD (e.g., Karki et al., 2011). However, despite all this effort at high pressures and temperatures, to date, no experiment nor simulation has been done on feldspars in the low‐pressure and high‐temperature region, which is of interest for the evolution of the protolunar disk.

Here we investigate the two alkali feldspar end‐members, NaAlSi_3_O_8_ and KAlSi_3_O_8_, in the low‐density region by performing FPMD simulations. We compute thermodynamic data as well as transport and structural properties over a wide range of densities and temperatures that are relevant for the synestia or the protolunar disk, as suggested by smooth particle hydrodynamics (SPH) simulations.

## 2 Material and Methods

### 2.1 Computational Parameters

We perform FPMD simulations as implemented in the Vienna ab initio simulation package (VASP) (Kresse & Furthmüller, 1996a, 1996b; Kresse & Hafner, 1993; Kresse & Joubert, 1999). We use the projector augmented wave (PAW) formulation (Blöchl, 1994) of the density functional theory (DFT) (Hohenberg & Kohn, 1964; Kohn & Sham, 1965; Mermin, 1965) to compute energy and forces, with the Perdew‐Burke‐Ernzerhof parametrization of the generalized gradient approximation (Perdew et al., 1996) for the exchange correlation term. We employ an energy cutoff of 550 eV for the plane waves and of 800 eV inside the augmentation sphere; we sample the reciprocal space in the Gamma point. Simulations are performed in the canonical NVT ensemble where the temperature (*T*) is controlled by the Nosé‐Hoover thermostat (Hoover, 1985; Nosé, 1984) around an average fixed value, and the volume (*V*) and the number of particles (*N*), are kept constant. At each time step the energy is converged to 1e−3 eV, which corresponds to at least seven representative digits in the absolute value of the energy.

We model the feldspar end‐members in cubic cells containing 208 atoms (16 formula units) and 1,024 or 1,152 electrons for the Na‐feldspars and K‐feldspars, respectively. A cubic cell of 14 Å side (volume of *V*_0_ = 2,744 Å^3^) corresponds to a density of 2.54 g cm^−3^ for the Na‐feldspar and of 2.69 g cm^−3^ for the K‐feldspar. The size of our simulations cells is typical for such FPMD studies on silicates. The relatively small number of atoms is compensated by the long trajectories, which allows for a good sampling of the configurational space. Chemical species have the time to form and break several times during the same simulation. Convergence tests carried out using 416 atoms for two temperature‐density couples (5000 K‐2.1 g cm^−3^ and 4000 K‐1.2 g cm^−3^) give a pressure difference of less than 0.12 GPa and a difference in the internal energy of less than 10 meV/atom between the two cell sizes. The pair distribution functions are invariant when changing between the simulations cells with 208 and 416 atoms. The relative proportions of coordination polyhedra remain constant within 1–2% between the two cells.

Production simulations are performed in the 2000‐7000 K range and 1–6 g cm^−3^ range in order to bracket the critical temperature and density. This also spans both liquid and liquid‐gas regions. This corresponds to a *ρ*/*ρ*_0_ range of about 2.4–0.4, *ρ*_0_ being close to ambient density (corresponds to *V*_0_ = 2,744 Å^3^). We use a time step of 1 fs in all simulations above 4500 K and 1.6 g cm^−3^ and of 2 fs below these conditions. The initial liquid state is obtained by heating a static configuration up to 4000 K for 8 ps and thermalizing it for 2 ps. This time lapse is enough to equilibrate the velocities of the atoms and to reduce the energy fluctuations. The other temperatures of interest are reached with one or more heating/cooling steps of 1000 K during 1 ps. Then at all temperatures and pressures we let the fluids equilibrate for at least 1 ps. We record production runs of 15–20 ps length after the total equilibration, and we use the final state to compress or expand the cell in order to reach respectively higher or lower densities.

To increase the speed of calculations and be able to reach low densities down to 0.5 g cm^−3^, we use pseudopotentials, which require a lower plane wave energy cutoff, set to 370 eV. In this region, the production runs last about 4 ps, which is enough to estimate the global pressure and temperature. For the simulations above 3.5 g cm^−3^, we use hard pseudopotentials in order to reduce the overlap of electronic spheres, in particular for Na‐Na pairs. The energy cutoff for this set of pseudopotentials is 950 eV.

### 2.2 Postprocessing

The postprocessing was realized using the UMD package (Caracas et al., 2020). The reported values of the thermodynamic potentials, that is, pressure and internal energy, are arithmetic time averages over the entire simulation, the spread in values is given by the standard deviation and the statistical error on the mean is computed using the blocking method (Flyvbjerg & Petersen, 1989).

The radial distribution function (*g*(*r*)) is the primary tool to analyze the structure of the fluids. It gives the average number of atoms of Type B in a spherical shell of radius *r* and thickness d*r* centered around each atom of Type A, relative to the number of atoms at the same distance in an ideal gas at the same density. Mathematically, *g*_*AB*_(*r*) is defined as

(1) $$g_{AB}(r)=\frac{n_{B}(r)}{n_{B}^{ideal}(r)},$$

(2) $$n_{B}^{ideal}(r)=\frac{4\pi }{3}\rho_{B}\left({\left(r+dr\right)}^{3}-r^{3}\right),$$

(3) $$n_{B}(r)=\frac{1}{N_{A}\tau_{run}}\overset{\tau_{run}}{\underset{\tau =1}{\sum }}\overset{N_{A}}{\underset{A=1}{\sum }}\overset{N_{B}}{\underset{B=1}{\sum }}\Pi_{\left(r,r+dr\right)}(r_{AB}),$$

with $\rho_{B}=\frac{N_{B}}{V_{cell}}$ being the atomic density of Type B atoms in the simulation cell of volume *V*_*cell*_, *N*_*A*_, and *N*_*B*_ the number of atoms of Types A and B in the cell, *τ*_*run*_ the total number of time steps and Π_(*r*, *r* + d*r*)_(*r*_*AB*_) the gate function, which is equal to 1 if *r* ≤ *r*_*AB*_ < *r* + d*r* (*r*_*AB*_ being the distance between the center of Atoms A and B) and 0 else.

We use d*r* = 0.05 Å as the discretization step of the computed *g*(*r*). The first peak of the *g*(*r*) offers a good approximation to the average bond length, representing the highest probability bond length. The first minimum of the *g*(*r*) function yield the radius of the first coordination sphere. It is this radius that we use as threshold to define if two atoms are bonded or not: if their interatomic distance *r*_*AB*_ is smaller than this radius, they are considered to form a chemical bond. Note that the chemical bonds do not have a temporal minimum limit, but only a maximal spatial extent. As such the minimum lifetime of a bond is the time step of the simulation.

The integral over *g(r)* up to its first minimum yields the average coordination number. The coordinating polyhedra are defined by the atoms that are found inside the first coordination sphere. Actually, the average coordination number corresponds to the sum of the order of the coordinating polyhedra weighed by their relative concentrations. In a more general way, the chemical species can be defined as the largest chains of connected atoms. At high pressure, as the fluid is compact this actually corresponds to an infinite polymer.

The self‐diffusion coefficient, *D*, is obtained from the Einstein relation:

(4) $$D_{A}=\underset{t\to \infty }{\lim}\frac{1}{6t}\left\langle \frac{1}{N_{A}}\overset{N_{A}}{\underset{i=1}{\sum }}|\vec{r_{A,i}}(t+t_{0})-\vec{r_{A,i}}(t_{0}){|}^{2}\right\rangle ,$$

where the term between brackets is the mean square displacement (MSD) for a time window with width *t*. The first part of the MSD, typical of a few hundred femtoseconds, corresponds to the ballistic part of the transport and the second part to the diffusive part.

Finally, we characterize the shocked state of the feldspars, in terms of pressure (*P*), density (*ρ*), and internal energy (*E*), according to the Hugoniot equation:

(5) $$E-E_{0}+\frac{1}{2}(P+P_{0})\left(\frac{1}{\rho }-\frac{1}{\rho_{0}}\right)=0.$$

To obtain the Hugoniot, we perform simulations at several temperatures and find the volume for which the pressure and internal energy values satisfy the Hugoniot equation.

### 2.3 Finding the Critical Point

**Figure 1:**
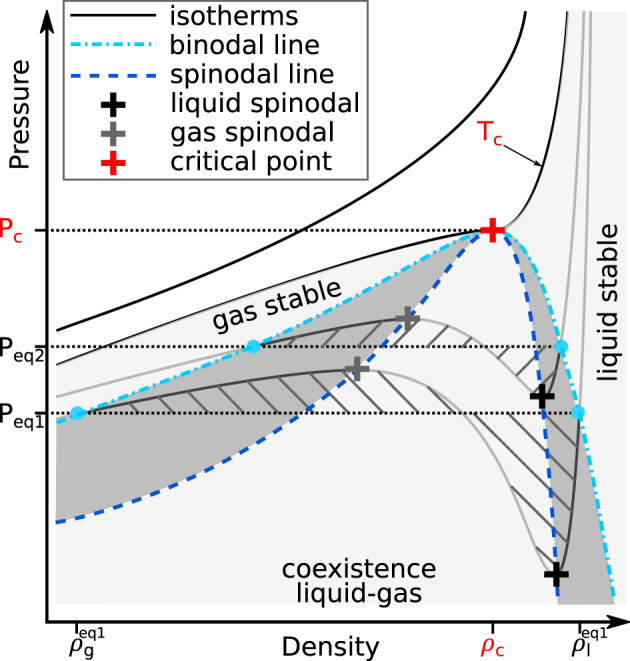
The analysis of the variation of the pressure as a function of density along different isotherms gives the stability domains for liquid, gas, and supercritical fluid. For a given isotherm, the extrema of the pressure correspond to the liquid and gas spinodal points; joining them yields the spinodal lines. The Maxwell equal‐area construction (hatched areas) yields the binodal line. At equilibrium, a liquid with density $\rho_{l}^{eq}$ is in equilibrium with a gas with density $\rho_{g}^{eq}$; they are both at the same equilibrium pressure *P*_*eq*_. Both lines have the critical point as a common maximum. The liquid and the gas are stable in the light gray areas and metastable in the dark gray areas, at respectively higher and lower density. The coexistence of gas and liquid is stable in the region between the two spinodals. The supercritical fluid is stable above the critical isotherm line.

Figure 1 shows a model for the pressure variation as a function of density for various isotherms. The local maximum and minimum of the isotherms define, respectively, the vapor and the liquid spinodal points. When these points are linked over all isotherms they construct the vapor and liquid spinodal lines. They both meet at the critical point. The states between the two spinodal lines are unstable as a single phase, that is, liquid or gas; only the mixture of the two is stable between the spinodals.

The Maxwell construction allows to find the equilibrium pressure and densities of the liquid and gas at a given temperature. Its derivation originates from equating the free energies of the liquid and the gas it can be constructed graphically from the pressure‐density relations along isotherms. In Figure 1, the equilibrium isobar is found when the area above it and below the *P*‐*ρ* curve on the gas side is equal to the area below it and above the *P*‐*ρ* curve on the liquid side. The intersection of the equilibrium isobar with the *P*‐*ρ* curve at a given isotherm defines the equilibrium liquid and gas density points, $\rho_{l}^{eq}$ and $\rho_{g}^{eq}$, respectively. They correspond to the limit of the stability domains: liquids are stable at densities higher than $\rho_{l}^{eq}$, and gases are stable at densities lower than $\rho_{g}^{eq}$. The spinodal points mark the limit of the metastability region. Liquids are metastable between the $\rho_{l}^{eq}$ and the liquid spinodal, gases are metastable at densities between the $\rho_{g}^{eq}$ and the gas spinodal. When connected at all temperatures, the equilibrium points define the liquid‐gas dome, also called the vapor‐liquid dome. Its importance comes from the role it plays during cooling and depressurization of a hot dense liquid or during condensation of a gas phase. Gas and liquid separate only when the temperature and density fall inside the dome; outside of it the thermodynamic stable state is monophasic: either as a liquid, a gas, or a supercritical fluid.

At low densities and low temperatures the configurational space of the vapor might not be fully sampled by the MD simulations, which prevents us from correctly describing the vapor structure and thus from using the Maxwell construction to obtain the full liquid‐gas dome. Nevertheless, we may explore the higher density region and compute the liquid spinodal points. We may sample the vapor region only in the vicinity of the critical point. Then we can estimate the position of the critical point. This method was successfully used in previous theoretical identification of critical points for SiO_2_ (Green et al., 2018) and Al (Faussurier et al., 2009). Binder et al. (2012) showed the presence of finite‐size effects in classical MD simulations using hard spheres interacting via Lennard‐Jones potentials. However to date, the location of the minimum in the pressure‐density curves is the best method for obtaining the spinodal curve (Faussurier et al., 2009; Green et al., 2018). The absence of minima in pressure above the critical temperature enforces the validity of the method.

## 3 Results

### 3.1 The Critical Points

We perform the calculations along several isotherms, ranging from 2000 K, corresponding to a hot magma, up to 7000 K, corresponding to the supercritical fluid. At each temperature we start at high density and decrease the density of the melt by expanding the volume of the simulation box. Depending on the temperature, we reach ambient pressure at densities of around 2.2 g cm^−3^ at 2000 K, 1.9 g cm^−3^ at 3000 K, 1.7 g cm^−3^ at 4000 K, 1.5 g cm^−3^ at 4500 K, and 1.3 g cm^−3^ at 5000K for the Na‐feldspar, and of around 1.9 g cm^−3^ at 3000 K, 1.6 g cm^−3^ at 4000 K, and 1.3 g cm^−3^ at 4500 K for the K‐feldspar. As we perform simulations at lower densities we go into extension regime and the pressure drops below 0, where the melts are metastable (i.e., the dark gray area in Figure 1).

The general equation of states (EoS) describing the liquid, liquid+vapor, and vapor states is the van der Waals EoS, which is a third‐order expansion of pressure in terms of density, spanning the entire liquid and gas stability regions. As we do not sample the vapor state we approximate the *P*‐*ρ* variation around the liquid spinodal with third‐order pressure‐density polynomials. We perform the fits along each isotherm. The minima of these curves show the position of the liquid spinodals as defined in the methodology section. The position of the critical point lies in density between the gas and the liquid spinodals and in temperature between the last isotherm that shows minima and maxima and the first isotherm that shows a monotonous decrease of pressure.

**Figure 2:**
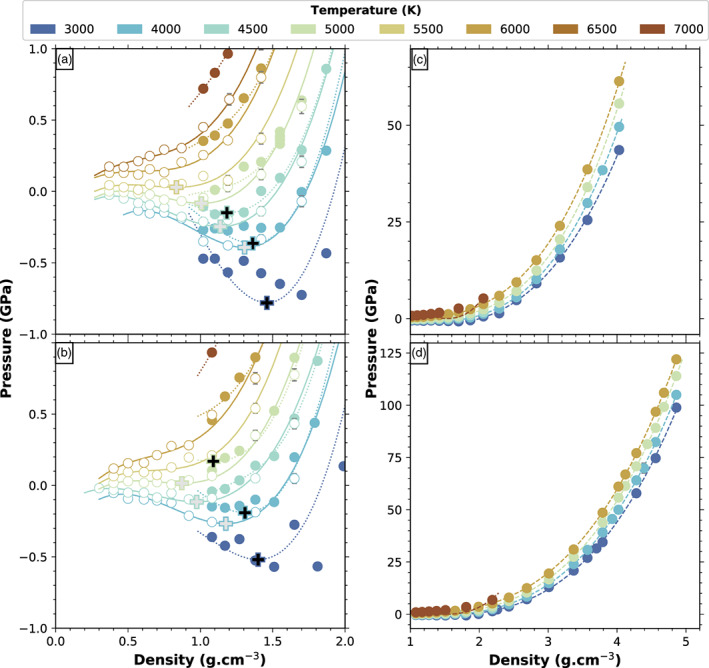
Computed pressure as a function of density around the spinodal part of the phase diagram for the (a) Na‐feldspar and (b) K‐feldspar end‐members and their respective view on the complete density range studied here, (c) and (d). Colors indicate temperature. Open symbols indicate values obtained with pseudopotentials which require a lower plane wave energy cutoff than those used to obtain the data set of solid symbols. Solid and dotted lines represent the third‐order polynomial curve fits corresponding to the respective two sets of data. The open and solid crosses indicate the liquid spinodals corresponding to the minima of the two sets of curves, respectively. The dashed curves in (c) and (d) are third‐order Birch‐Murnaghan equations of state fitted to our data. The statistical errors on the mean are included in the size of the points. For numerical data, the reader can refer to Data Sets S1–S5 in supporting information.

The Na‐feldspar shows pressure minima along isotherms up to 5500 K. The K‐feldspar shows minima for isotherms up to 5000 K. In both cases, the minima are less and less pronounced as the isotherms approach the critical temperature. For these temperatures we extend the simulations to low‐enough densities to observe a local maximum, corresponding to the gas spinodal (Figures 2a and 2b). There are no local minima for the 6000 K isotherm and above for the Na‐melt, and for the 5500 K and above for the K‐melt. Along these isotherms the pressure only decreases monotonously. As the liquid and the gas spinodal converge into the critical point, the liquid and the gas spinodals on the last isotherm bracket the density of the critical point. The isotherms with and without local minima and maxima bracket the temperature of the critical point. Hence, using our fitted values in the polynomial form, we obtain for the Na‐feldspar the critical point in the 0.5–0.8 g cm^−3^ and in 5500–6000 K range. For the K‐feldspar the critical point lies in the 0.5–0.9 g cm^−3^ and 5000–5500 K range.

Recently, two studies computed the critical point of SiO_2_ (Green et al., 2018) and MgSiO_3_ (Xiao & Stixrude, 2018) using FPMD. They found a supercritical temperature located between 5000 and 6000 K for SiO_2_, and around 6600 ± 150 K for MgSiO_3_, and a supercritical density respectively around 0.5–1.0 g cm^−3^ and 0.48 ± 0.05 g cm^−3^. The critical point of silica has been estimated before by Melosh (2007) to 5400 K and 0.55 g cm^−3^ based on thermodynamic calculations from ANEOS. The critical temperatures for two feldspar systems that we report here are similar to the critical temperature of SiO_2_ but all lower than the critical temperature of MgSiO_3_. The differences might arise from the degree of polymerization of the silica in the fluid.

Figure 2 shows the variation of pressure as a function of density for the Na‐feldspar and K‐feldspar end‐members along all computed isotherms. The two sets of pseudopotentials yield a slightly different position of the liquid spinodal at low temperatures. As we approach the critical point these differences considerably decrease. The statistical errors on the mean are included in the size of the points. Due to the large number of steps in each simulation runs, we obtain small uncertainties for the mean pressure even at very low densities. These values, of the order of 10^−3^–10^−2^ GPa, are smaller or about the same order of magnitude than those obtained by Green et al. (2018) who used the same method to estimate them. The spread of the pressure values during the simulation is about 1.6 GPa, which is typical for FPMD. The supporting information references the numerical results for pressure, temperature, density, and internal energy in Data Sets S1–S5.

### 3.2 Compressibility and Structure of the Fluids

In the one‐phase region, for pressures above 0, we fit third‐order Birch‐Murnaghan equations of states to the pressure‐density points along the 3000, 4000, 5000, and 6000 K isotherms. They are shown in Figures 2c and 2d. Table 1 shows the results and the comparison to existing data in the literature, both experimental (Lange, 2007; Tenner et al., 2007) and calculated (Bajgain & Mookherjee, 2020). The *ρ*_0_ values extrapolated from our simulations using a second‐order polynomial are 2.26 g cm^−3^ at 1373 K for NaAlSi_3_O_8_, that is, about 2.7% smaller than the experimental values (Lange, 2007).

**Table 1:** Parameters of Third‐Order Birch‐Murnaghan Equation of State Fitted to Our Computed Pressure‐Density Values at High Temperature and Comparison With the Available Experimental Data or Theoretical Results (FPMD) From the Literature

	Temperature	*ρ*_0_	*K*_0_		
	(K)	(g cm^−3^)	(GPa)	$K_{0}^{\prime }$	Source
NaAlSi_3_O_8_	298	2.615	56.4	3.9	crystalline albite, Tenner et al. (2007)
	1373	2.326	17.5	11	liquid, Tenner et al. (2007)
	2500	2.31	12	5.3	FPMD, Bajgain and Mookherjee (2020)
	3000	2.01	11	4.9	
	4000	1.82	6.5	5.8	
	5000	1.3	0.8	11	
	6000	0.9	2 × 10^−3^	7 × 10^2^	
KAlSi_3_O_8_	295	2.554	57	4	crystalline sanidine, Lange (2007)
	1473	2.298	15.8	12	liquid, Lange (2007)
	3000	1.8	5	6.5	
	4000	1.69	4.5	6.1	
	5000	1.3	0.9	9	
	6000	0.80	2 × 10^−3^	5 × 10^2^	

The isobaric expansivity $\alpha =\frac{1}{\rho }{\left.\frac{\partial \rho }{\partial T}\right|}_{P}$ and the isothermal compressibility $\beta =-\frac{1}{\rho }{\left.\frac{\partial \rho }{\partial P}\right|}_{T}$ are computed from our pressure‐temperature‐density points using the same method as described by Spera et al. (2009). The values are available in Data Sets S1–S5. At 3000 K and about 2.6 g cm^−3^, we obtain *α* = 4×10^−5^ K^−1^ for alkali feldspars, while Neilson et al. (2016) obtained about 5 × 10^−5^ K^−1^ with their classical MD simulation of liquid albite at the same conditions. An intermediate value of 4.7 × 10^−5^ K^−1^ was obtained by Stein et al. (1986) for liquid albite.

**Figure 3:**
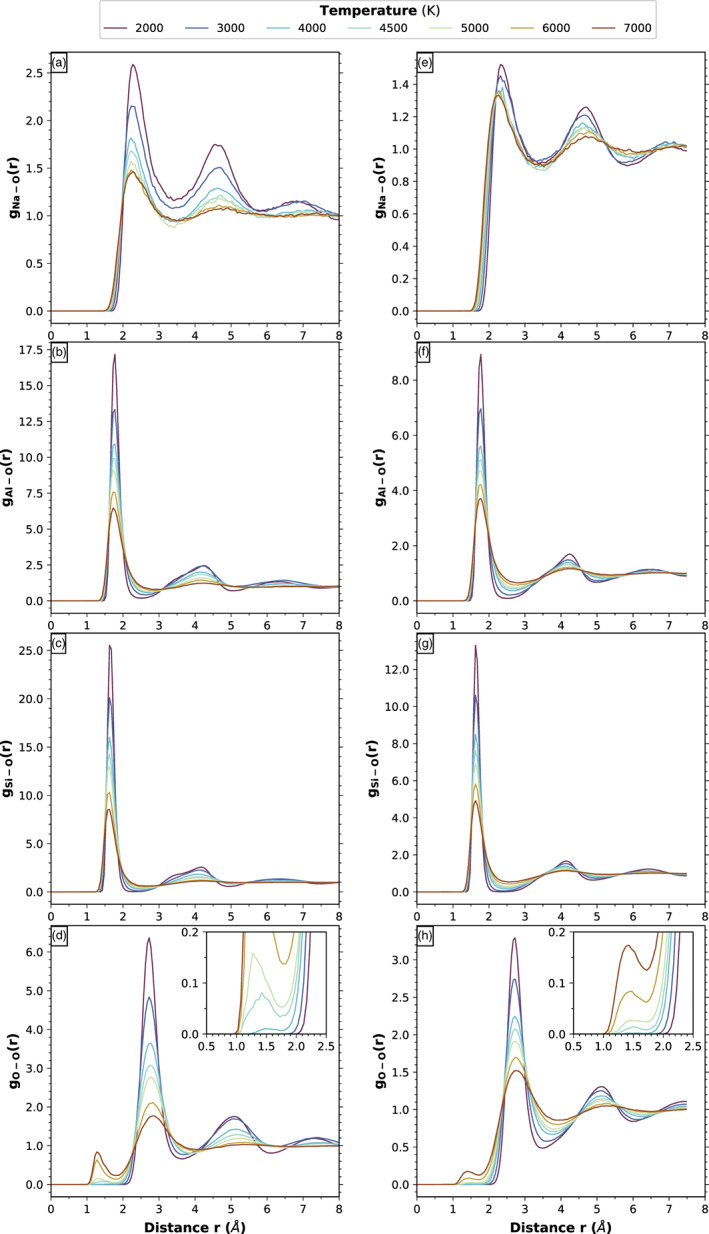
Pair distribution function of X‐O (X being Na, Al, Si, and O) in NaAlSi_3_O_8_ at 1.02 g cm^−3^ (a–d) and 2.06 g cm^−3^ (e–h). Colors indicate temperatures. The insert shows a zoom on the region 0.5–2.5 Å. The small peak located around 1.4 Å marks the presence of O_2_ molecules.

Figure 3 shows the pair distribution functions for the Na‐feldspar at two densities as a function of temperature stemming from our simulations. At 2 g cm^−3^ and 3000 K, conditions that are close to a hot magma at ambient pressure, the Si‐O, Al‐O, Na‐O, and K‐O average bond lengths are 1.64, 1.76, 2.31, and 2.79 Å, respectively. The T‐O bonds (T being Al or Si) vary weakly with both pressure and temperature; over 100 GPa pressure range the relative decrease is about 2%. However, the decrease of the Na‐O and K‐O average bond lengths over the same pressure range is on the order of 10% and 20%, respectively. In general the bond lengths in the liquid at 3000 K are comparable to the values recorded in the solids at ambient conditions. The radius of the first coordination sphere for the T‐O bonds decreases by about 10% over 100 GPa pressure range, while the radius for the M‐O (M being Na or K) follows the trend of the average bond lengths with a decrease of about 20%.

As tectosilicates, the structure of the solid feldspars is built of a polymerized framework of SiO_4_ and AlO_4_ tetrahedra with the alkali cations distributed orderly in the pores of this polymer. In the liquid state, the framework and polymerization are still present, but the dominant coordination changes as a function of both pressure or density and temperature.

**Figure 4:**
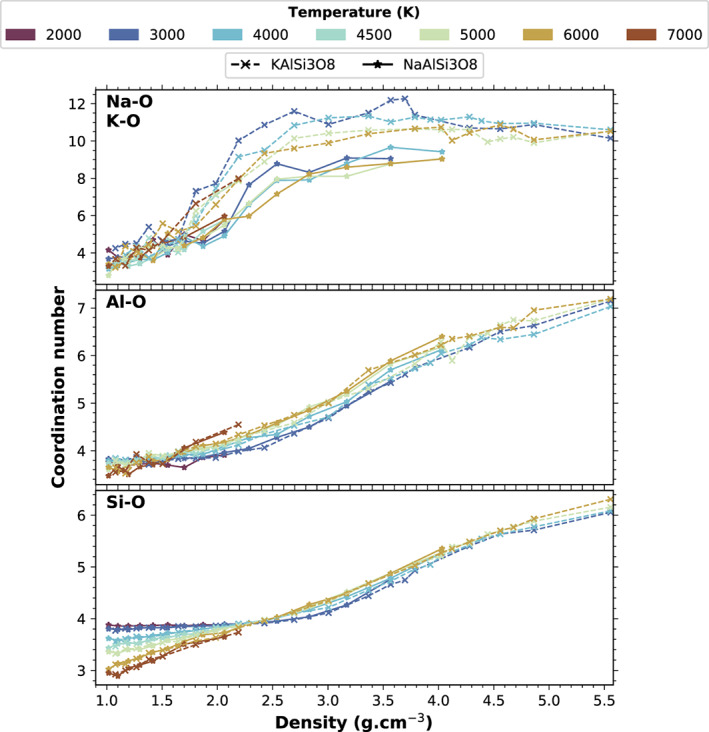
Average coordination number of Na, K, Al, and Si by O for the Na‐feldspar (stars and continuous lines) and K‐feldspar (crosses and dashed lines). At ambient conditions the solid or glass feldspars have NaO_6 − 9_, KO_9_, AlO_4_, and SiO_4_ coordination polyhedra (Jackson et al., 1987; Muller et al., 1993; Ribbe, 1984; Xue & Stebbins, 1993). Colors indicate temperature.

The average coordination number of Si and of Al by O in the computed fluids is similar for the two feldspar end‐members (Figure 4). For Si, it increases from about 3.8 at 2.2 g cm^−3^ to more than 5 above 4 g cm^−3^; it is only weakly dependent on temperature. At lower densities the dependence is stronger with temperature: At 1 g cm^−3^ it decreases from 3.8 at 3000 K to 2.9 at 7000 K. For Al, the coordination increases monotonously from about 3.7 at 1 g cm^−3^ to about 6 above 4 g cm^−3^ (corresponding to about 50 GPa). The spread of coordination numbers due to temperature is less than 0.5 units between 3000 and 6000 K at all densities. The coordination number of Al by O is larger than the coordination number of Si by O at all densities and temperatures.

**Figure 5:**
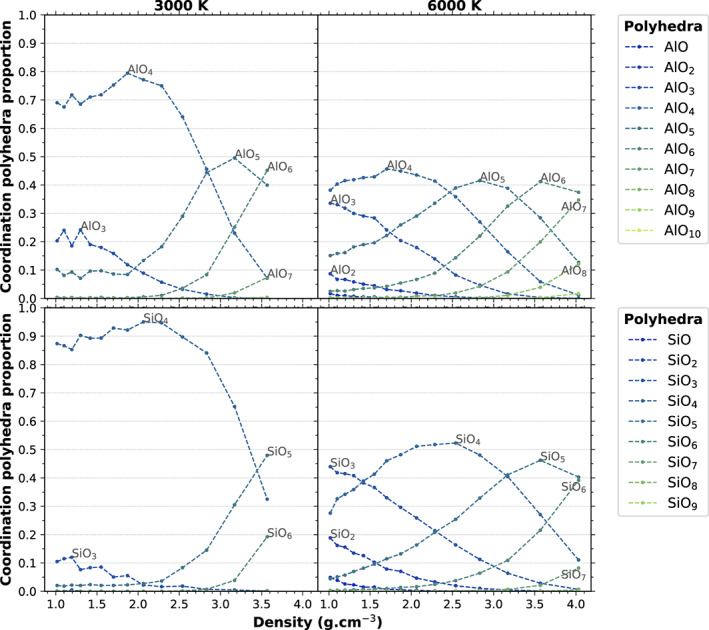
Relative proportion of SiO_*x*_ and AlO_*x*_ coordination species in NaAlSi_3_O_8_ for 3000 and 6000 K as a function of density.

**Figure 6:**
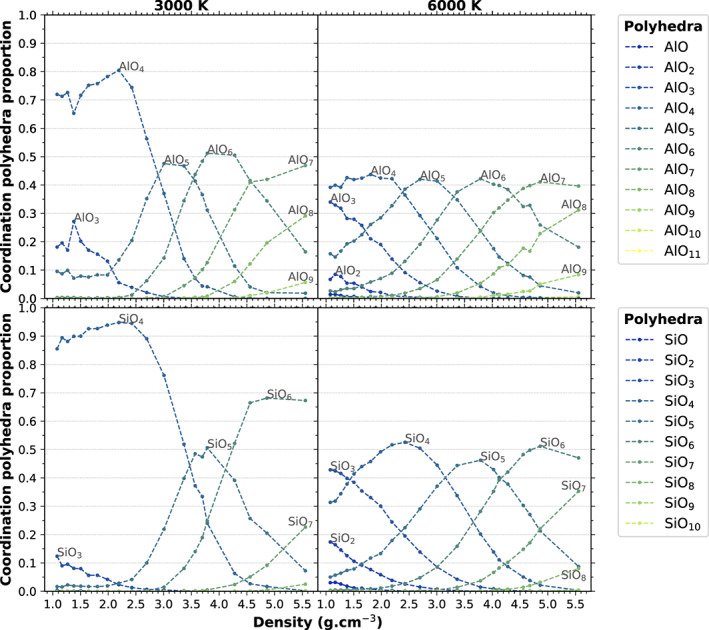
Relative proportion of SiO_*x*_ and AlO_*x*_ coordination species in KAlSi_3_O_8_ for 3000 and 6000 K as a function of density.

In terms of actual species rather than average coordination numbers, the two feldspar systems behave in a similar way to other silicate melts (Karki et al., 2018; Solomatova & Caracas, 2019). Figures 5 and 6 show the proportion of the SiO_*x*_ and AlO_*x*_ species in the NaAlSi_3_O_8_ and KAlSi_3_O_8_ systems, respectively, for two relevant temperatures below (3000 K) and above (6000 K) the critical temperature as a function of density. In general, the dominant species contains more O atoms for Al than for Si. At densities below 2.2 g cm^−3^, the amount of undercoordinated Si and Al, that is, SiO_*x*_ and AlO_*x*_ species with *x* < 4, increases with decreasing density and increasing temperature. This comes from the decrease of the coordination of cations by oxygen at the interface between the voids and the melt, as the system becomes metastable at lower densities.

At densities above 2.2 g cm^−3^, under compression, the melt is dominated by SiO_4_ tetrahedra up to about 25 GPa at 3000 K and 20 GPa at 6000 K. SiO_5_ is present in the melt at low pressures at all temperatures. SiO_6_ appears around 10 GPa at 3000 K and 5 GPa at 6000 K; it is the dominant species above 70 GPa at 3000 K and above 50 GPa at 6000 K.

**Figure 7:**
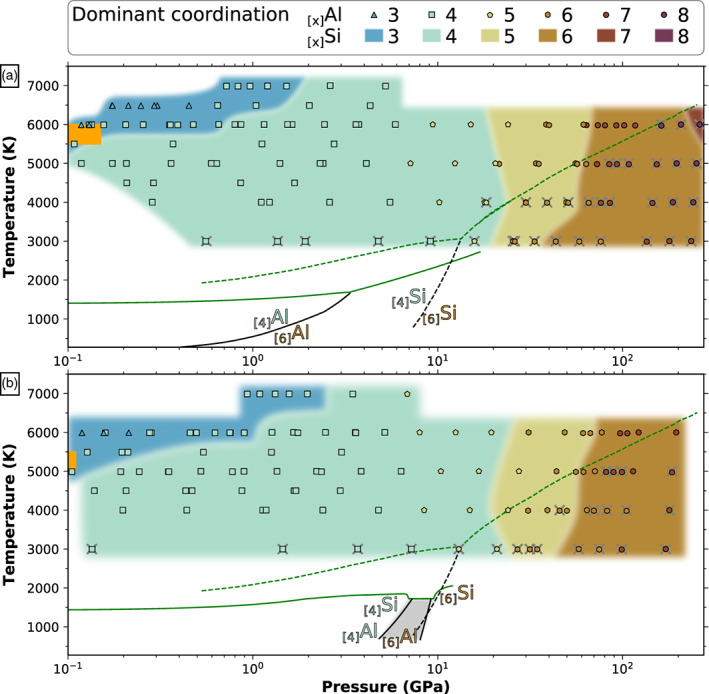
Changes of the major coordination of T by O (T being Al, Si) for (a) NaAlSi_3_O_8_ and (b) KAlSi_3_O_8_ compositions in the temperature‐pressure projection plane. Background color indicate the number of O atoms in the SiO_*x*_ coordination polyhedra that dominates the structure of our computed melt. The shape and color of symbols indicate the number of O atoms in the AlO_*x*_ coordination polyhedra that dominates the structure of our computed melt. Dashed lines are solidus (green) and coesite‐stishovite phase transition (black) for silica from Zhang et al. (1996) and Tsuchiya and Tsuchiya (2011). Solid lines are solidus (green) and solid‐solid phase transitions (black) for alkali feldspars (and jadeite above 2 GPa) from Bell and Roseboom (1969), Litvin and Gasparik (1993), Newton and Smith (1967), Urakawa et al. (1994), Lindsley (1966), and Akaogi et al. (2004). The subsolidus coordination indicated in this diagram (denoted _[4]_T and _[6]_T for coordination number of T by O of 4 and 6, respectively) are for the crystalline phases. They may differ from the glasses coordination. The gray area indicate the region of K_2_Si_4_O_9_ wadeite‐type mineral, with Si fourfold and sixfold coordinated to O. The orange rectangle indicate the estimated location of the critical point as computed in section 3.1.

Figure 7 shows the change in the major coordination of Si and Al by O as a function of pressure and temperature and the comparison between the fluids and the solids (crystalline forms). The melts are characterized by a series of structural changes where the dominant coordination goes as TO_4_→ TO_5_→ TO_6_→ TO_7_ (→ TO_8_), T being Si or Al. This transition series follows closely the changes recorded in the solids, but the sequence is generally shifted toward higher pressures. The increasing temperature in the fluid shifts to larger pressure the change in dominant coordination. However, the solids do not show fivefold, sevenfold, or eightfold coordination.

The lifetime of the coordination polyhedra underlines the long‐lasting nature of the 4‐fold coordination species SiO_4_ and AlO_4_ in the melts below the critical temperature. For example at low densities and 3000 K, the SiO_4_ tetrahedra live up to 10 ps, and at 4000 K up to almost 4 ps. Increasing temperature leads to decreasing the maximum lifetime of all coordination polyhedra and extending the tail of lifetimes, as the bonds form and break with higher frequency. The SiO_*x*_ species tend to live longer than the AlO_*x*_ species at the same density and temperature. Figures S1a and S1b in the supporting information display the lifetime of each type of coordination polyhedron at 3000 and 6000 K for respectively Na‐feldspar and K‐feldspar melt at about 2.2 g cm^−3^.

Na and K act as interstitial cations in the large Si‐Al‐O polymer that constitutes the framework of the melt. For this reason their coordination polyhedra by O show a much larger variability than their equivalent Si and Al. Also, the lifetime of the NaO_*x*_ and KO_*x*_ coordination polyhedra (Figure S2 in the supporting information) is considerably shorter than the SiO_*x*_ and AlO_*x*_.

### 3.3 Volatilization

The largest chemical species found in the simulations involve forming chains and rings of alternating cations and oxygens, in …‐oxygen‐cation‐oxygen‐cation‐oxygen‐… sequences. At high density all 208 atoms are connected in one infinite cluster, like an infinite polymer, which constitutes the liquid.

**Figure 8:**
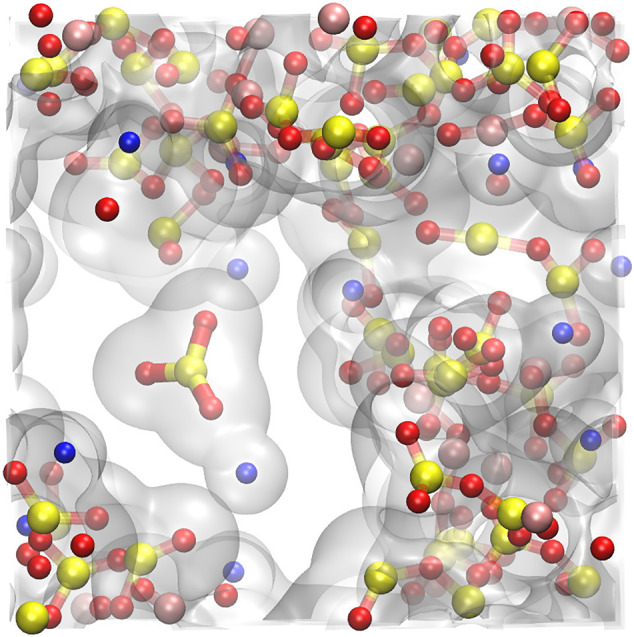
Snapshot of the iso‐electronic density surface at 0.01 e/Å^3^ in the simulation cell at 4000 K and 1.02 g cm^−3^ created with VMD (Humphrey et al., 1996). We see a clear bubble with a NaSiO_3_ cluster inside. Colors indicate elements: red = O, yellow = Si, pink = Al, and blue = Na.

**Figure 9:**
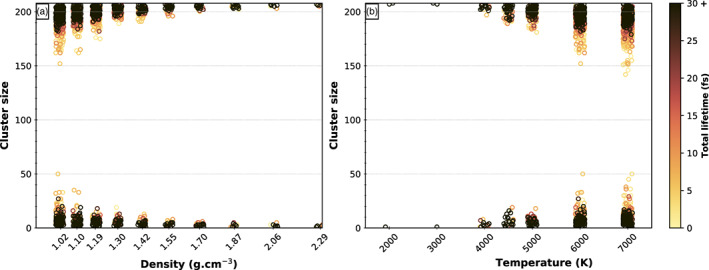
The size of all individual clusters of atoms in NaAlSi_3_O_8_ simulations as a function of (a) density at 6000 K or (b) temperature at 1.02 g cm^−3^ follow a bimodal distribution. The larger clusters polymerize to form the melt and the smaller clusters are separated, forming the gas phase. The analysis of the speciation takes into account the periodic character of the simulations. Each circle represents an individual cluster of the corresponding size (*y* axis). Color scale indicates the lifetime of each cluster. Clusters in the gas with size larger than 13 atoms have lifetimes shorter than 30 fs, with the large majority even shorter than 10 fs. In all simulations there is a stable liquid part, with big cluster above 200 atoms living more than 30 fs.

At low densities and below the critical temperature, long‐lasting cavities appear in the simulations, where isolated atoms or clusters of atoms may freely float inside. They represent the nucleating gas bubbles. Figure 8 shows a typical snapshot of the simulation cell of NaAlSi_3_O_8_ at 4000 K and 1.02 g cm^−3^ with the electronic density isosurface at 0.01 e/Å^3^ drawn to indicate the different clusters. We see a large interconnected atomic polymer, forming the melt, and one NaSiO_3_ cluster isolated from the rest of the atoms; this represents one of the first gas components in the nucleating bubbles. The distribution of the size of the atomic clusters is bimodal: the larger polymerized melt that surrounds the cavities and the smaller atomic clusters that populate these cavities (Figure 9). We notice that in all simulations, most of the species above 200 atoms or below 13 atoms live more than 30 fs.

In the melt at 4000 K, a limited number of species appear in the gas bubbles, with average lifetimes between 60 and 300 fs. The gas is dominated by free Na and K cations making up for at least half of the gas. Second in importance are small volatile species, like O_2_, SiO, and SiO_2_. In both feldspars Al is present in the gas phase only as traces as AlO or more complex species involving K, Na, Si, and O. The volatilization of both feldspars is incongruent, mainly due to this lack of Al in the gas phase (see Figure S3 in the supporting information). Between 1.4 g cm^−3^ and 1 g cm^−3^ the molar proportion of K and Na in the gas almost double with respect to the total available K and Na in the system, while Al remains almost entirely in the melt.

**Figure 10:**
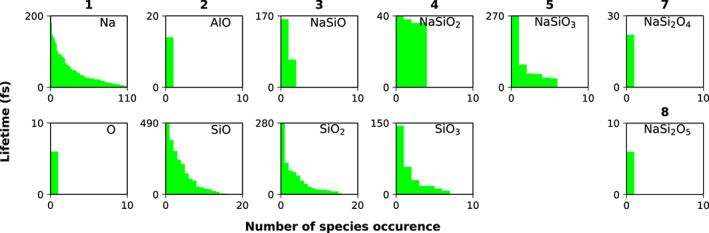
Lifetimes of isolated atomic clusters with less than 13 atoms at 1.02 g cm^−3^ and 4000 K.

**Figure 11:**
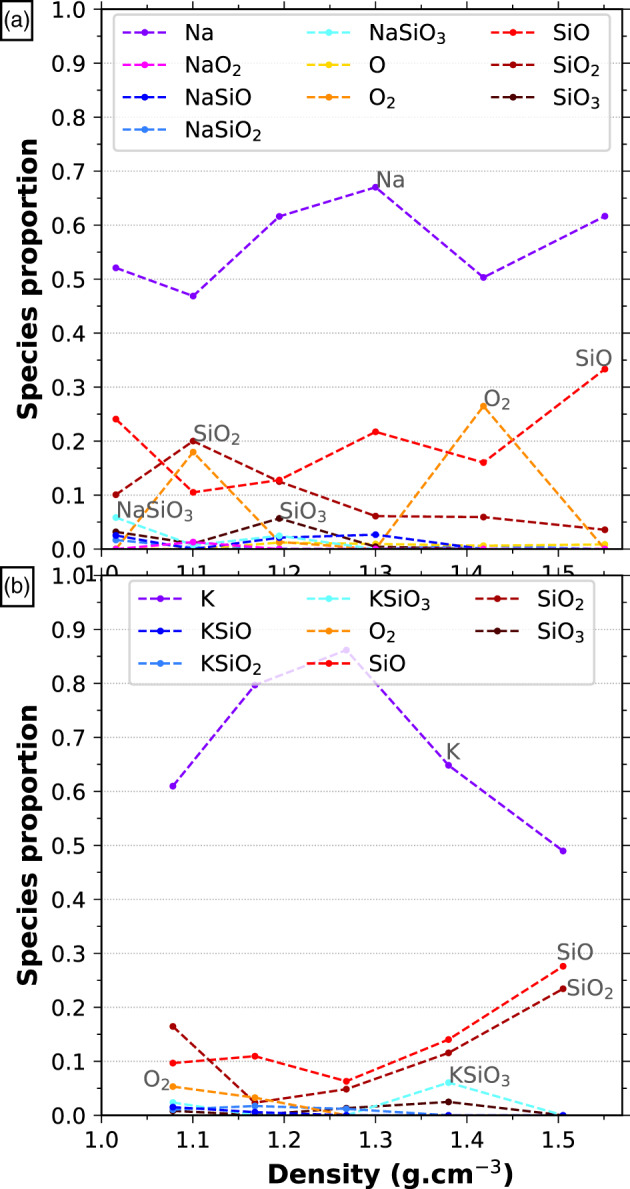
Proportion of all the isolated clusters of length less than 13 atoms as a function of density at 4000 K for (a) Na‐feldspar and (b) K‐feldspar end‐members. Species that represent more than 5% of all the gas species for at least one density point are labeled in the graph. The other species, found in trace amounts, are the following (a) AlO, NaO, Si_2_O_*z*_ (*z* ∈ [3, 5]) and some species among Na_*w*_Si_*x*_O_*z*_ (*w* ∈ [1, 3], *x* ∈ [1, 2], *z* ∈ [1, 5]); (b): O, Si_2_O_3_, Si_2_O_4_, K_*w*_AlO_2_ (*w* ∈ [0, 1]) and some species among K_*w*_Si_*x*_O_*z*_ (*w* ∈ [1, 3], *x* ∈ [1, 3], *z* ∈ [3, 7]).

In the supercritical fluid at 6000 K there are no cavities, so we cannot talk about a gas phase: The species are all in a single fluid phase. The speciation becomes more diverse, with many more clusters being composed of 4 to 13 atoms. These clusters have short lifetimes and high mobility, inducing density fluctuations in the fluid over short time scales, which is characteristic of supercritical fluids. But at larger time and length scales the melt is homogeneous. In parallel there are fewer isolated Na but more isolated O atoms. The O_2_ molecules live up to 550 fs. Figure 10 illustrates the lifetime of the isolated species with less than 13 atoms in the simulation of Na‐feldspar end‐member at 1.02 g cm^−3^ and 4000 K. Figure 11 shows the relative proportion of species with less than 13 atoms. Visscher and Fegley (2013) obtained the same major species using the MAGMA code (Fegley & Cameron, 1987; Schaefer & Fegley, 2004), but their species are more abundant, especially O_2_ which is present in their estimations at all temperatures.

### 3.4 O_2_ Behavior

A characteristic feature occurring at high temperatures is the presence of free O_2_ molecules in the fluid. Figures 3d and 3h shows the evolution of the pair distribution function of O‐O with temperature for two representative densities: 1.0 g cm^−3^, which is below the liquid‐gas boundary density and 2.1 g cm^−3^, which is in the fully fluid region. At low temperatures, the pair distribution function of O‐O displays one main peak located around 2.7 Å, which represents the position of oxygens sharing the same edge of polyhedra around the Al or Si cations. The peak is found at all densities and temperatures and its position varies from 2.5 Å at high densities to 2.8 Å at low densities. At high temperatures the pair distribution function exhibits a second smaller peak located around 1.4 Å. This represents the O‐O bond in a O_2_ molecule. This peak can be seen at almost all densities above 3.5 g cm^−3^ only for temperatures higher than or equal to 4500 K, and only at very low densities at 4000 K. Its position varies from approximately 1.5 Å around 3 g cm^−3^ to 1.3 Å at 1.0 g cm^−3^.

We find O_2_ molecules in both feldspar compositions studied here. This was also observed in previous simulations on pure SiO_2_ (Green et al., 2018) and MgSiO_3_ (Xiao & Stixrude, 2018). These consistent results suggest that there is potential degassing of O_2_ from the silicate melt at high temperatures, independently of the composition of the melt. The process starts between 4000 and 4500 K.

According to Green et al. (2018), O=O pairs in silica survive for less than one vibrational period. For feldspars we observe speciation lifetimes on the order of several hundreds of femtoseconds, which is considerably longer than the vibrational period. It is possible that cations other than Al and Si, like Na and K, reduce the polymerization of the melt at these conditions and then enhance the formation and survival of O_2_ groups.

### 3.5 Transport Properties

The MSD of all atoms at every temperature and density display a ballistic part, which corresponds to the conservation of the velocity of atoms after collisions, which last on the order of 100 fs, followed by a fully diffusive part, which corresponds to the scattering of the velocity of atoms after collisions (Figures S4 and S5 in the supporting information).

Along the 2000 K isotherm we observe a strong decoupling between the diffusion of Na or K and the diffusion of Si, Al, and O, with more than 1 order of magnitude difference at 1.0 g cm^−3^. At 3000 K and about 1 GPa, all atoms travel considerably shorter distances on the order of 7–8 and 2 Å for respectively K, Na, and Al, Si, O over the same amount of time. At 5000 K and densities around 1 g cm^−3^, after 8 ps Al travels around 12–14 Å while Si and O travel 16–17Å, and Na, K travel, respectively, 25 and 22 Å. At about 2.2 g cm^−3^, the traveled distances over the same time decrease down to 9 Å for Si, 9–10 Å for Al, 10–11 Å for O, 12 Å for K, and 16 Å for Na. There is no abrupt change in the mobility of the atoms during the passage from the hot magma to the supercritical fluid.

Figures S4 and S5 in supporting information show the MSD for all the temperatures and densities studied here. They are almost linear with respect to time, with some exceptions for Na and K especially at low density. Since free cations move further away than big clusters of atoms, these variations in the curve slopes can be explained by the volatilization of isolated Na and K. Indeed, this is seen in the analysis of the MSD for individual atoms, as the free cations have larger MSD and distinguish themselves from the cations in the melt.

**Figure 12:**
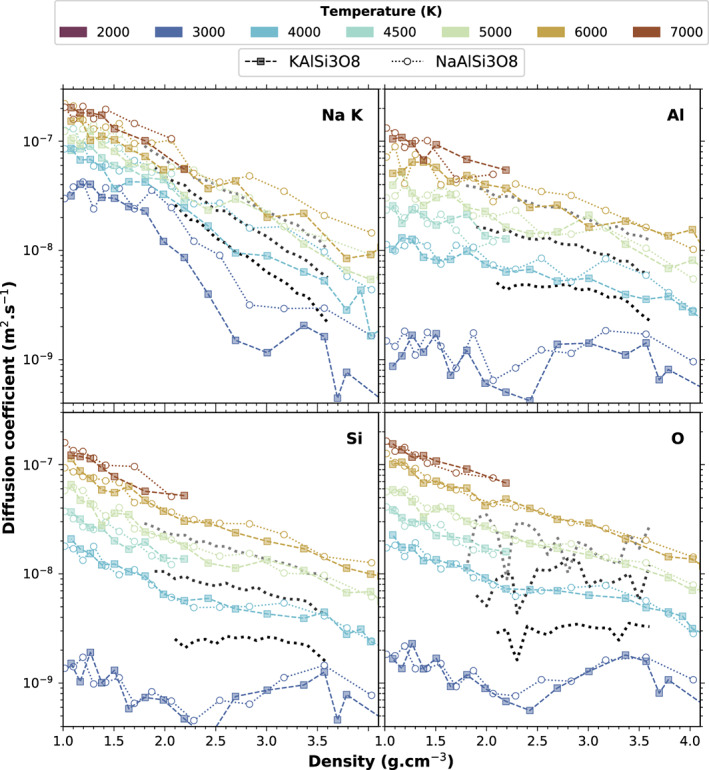
Self‐diffusion coefficients for every element as a function of density. The black to light gray lines are theoretical results from Neilson et al. (2016) on NaAlSi_3_O_8_ at approximately 3000, 4000, and 5000K˙.

The slope of the MSD yield the self‐diffusivity coefficients, which are shown in Figure 12 for Al, Si, O, and the cations Na and K as a function of density for temperatures between 3000 and 7000 K. Na and K are always the most diffusive elements at low densities. Their diffusion coefficients decrease by about 1 order of magnitude over the 2.0–4.0 g cm^−3^ density range. The self‐diffusivities of Al, O, and Si are similar along each isotherm, resulting from the polymerized character of the melt. Their diffusion coefficient is about half an order of magnitude smaller than for the Na and K cations, which occupy the interstitial space between the silica and alumina polymers. The difference in diffusion coefficients correlates well with the difference in lifetimes of coordination polyhedra of the different species, the NaO_*x*_ and KO_*x*_ species having shorter lifetimes than the SiO_*x*_ and AlO_*x*_. The difference between isotherms is reduced when the temperature increases. At high temperature and low density, the self‐diffusivity of every element tends toward 1–2 × 10^−7^ m^2^ s^−1^.

**Figure 13:**
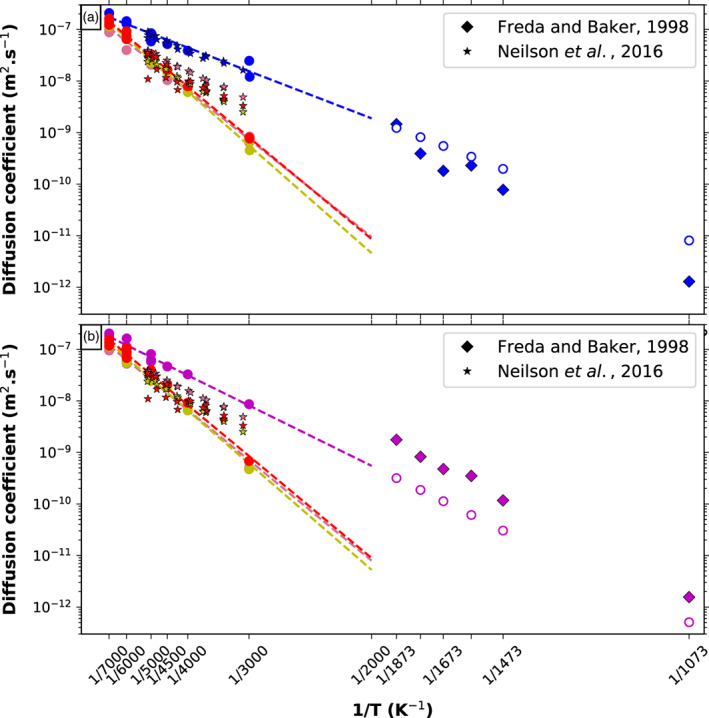
Self‐diffusion coefficients for (a) Na‐feldspar and (b) K‐feldspar end‐members at 1 GPa as a function of the inverse temperature (filled circles), and fit of the Arrhenius law (dashed lines). Colors indicate elements: red = O, yellow = Si, pink = Al, blue = Na, and purple = K. The stars indicate results from a classical MD simulation on molten albite (Neilson et al., 2016), and diamonds are experimental results on albite‐orthoclase melts (Freda & Baker, 1998). Empty circles are the extrapolated diffusivities at the experimental temperatures using the Arrhenius fit of our data.

The values for diffusion coefficients that we obtain from our simulations are on the same order of magnitude as the theoretical values published previously on various other silicate melts. For example, *D*_*Si*_ at 4000 K and 1 GPa that we obtain in hot liquid feldspars is around 6 × 10^−9^ m^2^ s^−1^, compared to about 1 × 10^−8^ m^2^ s^−1^ in anorthite (de Koker, 2010), 1 × 10^−9^ m^2^ s^−1^ in silica (Karki et al., 2007), and 8 × 10^−9^ m^2^ s^−1^ in pyrolite (Caracas et al., 2019). An Arrhenius fit to the theoretical values of diffusion extrapolated to low temperatures, yields diffusion coefficients similar to experimental values obtained between 1000 and 2000 K in alkali feldspar melts (Freda & Baker, 1998). The agreement between extrapolated values and experimental results is better for Na than for K. Figure 13 shows our theoretical results at low pressure, compared to the available experimental data (Freda & Baker, 1998).

At 2000 and 3000 K for densities larger than about 1.7 g cm^−3^ diffusion is very sluggish for Al, Si, and O. Indeed, some of the simulations at low temperature are in the regime of undercooled melt, laying below the melting lines. Spera et al. (2009) suggested that in order to be accurate and meaningful, liquid MD simulations must be performed for temperatures above the computer glass transition, estimated around 2800 K for CaAl_2_Si_2_O_8_, a value that increases when the pressure increases. Other works (Harvey & Asimow, 2015) suggest that sufficient sampling of the configuration space is achieved when all atoms in a melt change at least one crystallographic site. For a polymerized silicate melt, this corresponds to a displacement of all Si atoms, the least diffusive species, by at least 3 Å according to the pair distribution function of Si‐Si, or a mean square displacement of at least 9 Å^2^ within the length of the production run. This value extends beyond the first coordination sphere of Si by Si. This distance can be traveled only by allowing for long simulation times, which is now tractable on the available computational resources. In the large majority our simulations are long enough to show a MSD of Si larger than 9^2^.

## 4 Behavior of a Feldspathic Crust During Impacts

The early part of the Hadean was dominated by impacts as the main phase of the accretion unfolded. Many could have generated partial melting and partial freezing episodes (e.g., Elkins‐Tanton, 2012), which in turn could have led to the formation of some primitive crust. The giant impact marked the end of this major part of Earth's history. From the subsequent protolunar disk the Earth condensed and differentiated into the central liquid core enveloped by the magma ocean, that is, the molten state of the entire mantle. The crust eventually separated from cooling of the magma ocean. In a terrestrial‐like planet, even if we cannot know precisely what would be the extent of the crust nor the temperature at the surface of the proto‐planet, feldspars must have been one of the major components of this crust, because of their buoyancy and their early position in the crystallization sequence.

**Figure 14:**
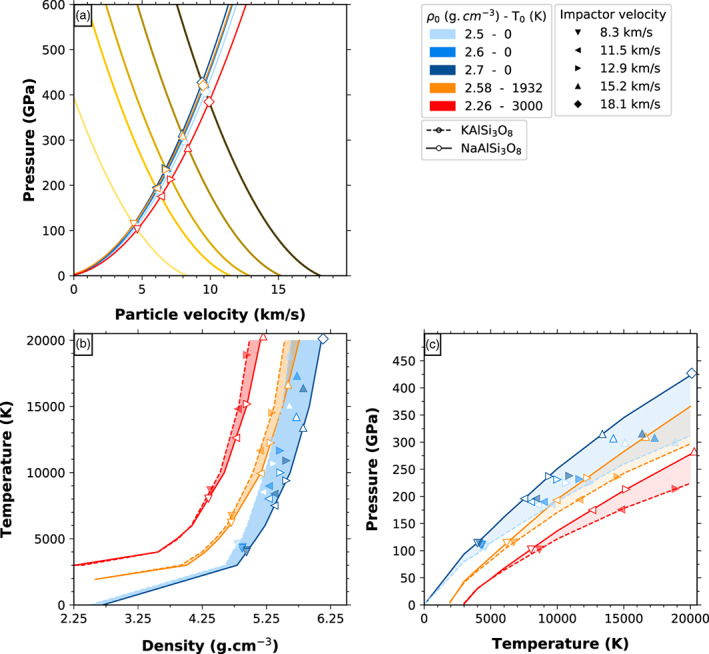
Peak shock conditions are obtained at the intersection between the cold Hugoniot curve of the impactor and the Hugoniot curves of the crust (a). For the crust we consider various initial temperatures and densities, represented by different colors. We consider several possible impactor velocities. The peak conditions are then represented with different symbol shapes in several projections: (b) temperature‐density and (c) pressure‐temperature. Open and filled symbols for the K and Na‐feldspar respectively. Numerical values for the Hugoniot curves and peak shock condition are available in supporting information Tables S1–S4.

In order to model the behavior of the major alkali feldspathic component of the crust during shock as generated by large impacts we build the Hugoniot equations of state, using our computed density‐pressure‐temperature points. Using the computed Hugoniot equations of state we can infer the shock state after large impacts for the Earth and the Moon crusts using the impedance match method (Forbes, 2012) presented in Figure 14a.

We consider three possible initial thermal states, which we denote as cold, warm and hot; for the cold state we further consider three densities at ambient pressure, corresponding to the range found in the continental crust. The Hugoniot equations of state obtained for the different initial states are represented in Figures 14b and 14c.

For the first scenario, temperatures are considered to be close to the ambient temperature of today. This case is relevant for shock experiments or crustal impacts on cold astronomical bodies, for example depleted of atmosphere. The crust is solid, made of feldspar crystals. As an extreme case, we infer a thickness of up to 50 km (current values are an average of about 30 km for the Earth and about 50 km on the Moon; Taylor & McLennan, 2008). According to the estimated variations of the pressure as a function of depth inside terrestrial planets (Warren, 1985), the pressure at the bottom of such a crust would be less than 2 GPa. This is translated in densities in the 2.5–2.7 g cm^−3^ range for the two feldspars. We consider three density points in this range, at 2.5, 2.6, and 2.7 g cm^−3^ and model the initial state using standard static calculations on ideal triclinic feldspar crystals. We find that the thickness of the crust or the possible presence of a shallow atmosphere have a weak effect on the Hugoniot equations of state (blue curves and areas in Figures 14b and 14c).

For the second scenario, we assume the surface temperature to be above the melting temperature of feldspars, as if a magma ocean has just crystallized. The melting temperature of feldspar varies with the composition. It ranges from 1383 K for Na composition to 1823 K for Ca composition (Bowen, 1913), and is around 1473 K for K end‐member (Lange, 2007). We choose to use 1932 K as initial temperature in order to compare our results with those obtained experimentally by Asimow and Ahrens (2010) on anorthite. This scenario is particularly relevant for the crystallization of the Moon. In a dry lunar magma ocean, feldspars float whereas they sink in a terrestrial wet magma ocean (Taylor, 1982). For this case the Hugoniot curve lies at higher temperatures and pressures than in the previous case (orange curves and area in Figures 14b and 14c).

For the third scenario, temperatures are similar to the surface of a hot magma ocean, as covered, for example, by a thick silicate atmosphere. The density of a liquid feldspar is much lower than its corresponding solid form. This effect combined with the thermal expansion of liquids leads to a density of 2.26 g cm^−3^ at a temperature of 3000 K in the case of the K end‐member (Lange, 2007). The calculated Hugoniot line is the highest in both temperature and pressure from all the three cases (red curves and area in Figures 14b and 14c).

Up to 5000 K the two feldspars have similar Hugoniot equations of state. At higher temperatures the pressure along the Hugoniot of the K‐feldspar deviates by up to 75 GPa above that of the Na‐feldspar and the temperature by about 2000 K above that of the Na‐feldspar. For both feldspars, the initial temperature has weak influence on the final temperature and pressure, but decreasing the initial density leads to considerably higher Hugoniot temperatures and lower pressures. We do not specifically calculate the Hugoniot equations of state beyond 20000 K since the pseudopotentials are missing electronic states that might be occupied at such high temperatures. However, we are able to provide an extrapolated estimate based on the computed lower temperatures (see Tables S1 and S2 in the supporting information).

For the impedance match method we make the approximation of planar waves generated by the impacts, which propagate in the two bodies after the initial shock. Then equating the pressure in the dynamic impactor and the static target yields the peak shock conditions.

We choose velocities for the impactor of 12.9, 15.2, and 18.1 km s^−1^ for the impact with the Earth and 8.3, 11.5, and 15.2 km s^−1^ for the impact with the Moon. These values correspond respectively to the first, second, and third quartiles of the Earth and Moon impactor velocities obtained on a basis of 1,487 impacts generated for the Earth in the work of Raymond et al. (2013) on planetary impacts during the late veneer (personal communication). We employ the formalism from Raymond et al. (2013) (Equations (3), (4), (5)) to compute the two‐body escape velocities along with the impact velocities on the Moon. We consider all the impactors to have a density of 3.0 g cm^−3^ regardless of their possible composition, as in Raymond et al. (2013), and 0 K temperature.

We obtain the peak shock conditions by intersecting the cold Hugoniot of the impactor with the various Hugoniot equations of the cold, hot, or molten feldspar crust (Figure 14a). The results are shown in Figures 14b and 14c for the various possible thermal and dynamical scenarios for both the target and the impactor (for numerical values see Tables S3 and S4 in the supporting information).

Our results show that for velocity impacts lower than about 10 km s^−1^ in a cold crust the peak temperatures would remain below 4500 K and pressures below about 100 GPa; at these conditions the crust would enter a premelting regime or might even melt. At impact velocities larger than 10 km s^−1^ but still in a cold crust, the peak conditions would exceed 7500 K in temperature and about 200 GPa in pressure. This suggests that the peak conditions of the impacts that produced the large basins on the Moon were energetic enough to have brought the crust into supercritical state. The lava then condensed along the liquid‐vapor equilibrium lines, which implies that a large part of the volatile components would have been lost into space.

In the case of early Earth, hot crust or local magma ponds likely existed and impacts into these structures would have had a very different outcome. In this case the temperatures can reach between 10000 and up to 30000 K depending on the impactor velocity. At these conditions the integrity of the crust would be completely erased, as all materials would melt and reach supercritical state. The crust would then be integral part of the protolunar disk, bringing a silica‐ and alkali‐rich contribution. This was the case of the Giant Impact.

## 5 Conclusions

We computed the critical point of alkali feldspars, using FPMD calculations. We find that two critical points lie at similar thermodynamic conditions: 0.5–0.8 g cm^−3^ and 5500–6000 K range for the Na‐feldspar, 0.5–0.9 g cm^−3^ and 5000–5500 K range for the K‐feldspar.

We determine a number of physical properties of the Na‐fluids and K‐fluids and find them remarkably similar. The speciation shows increasing coordination to be the favorite mechanism for accommodating compression. At low pressure, the melt is dominated by polymerized silica and alumina tetrahedra; a fivefold coordination component is already present at 0 GPa. As pressure increases, above about 10 GPa, SiO_6_ and AlO_6_ appear in the melt. Their lifetime is on the order of 30 fs at 3000 K and decreases drastically down to 15 fs at 6000 K. SiO_6_ becomes the dominant silica species above 70 GPa at 3000 K and 50 GPa at 6000 K. SiO_7_ species appear at around 40 GPa at 6000 K. In general Al shows a larger coordination than Si at any given pressures and temperatures.

Our FPMD simulation suggest that there is potential degassing of O_2_ from the silicate melt starting between 4000 and 4500 K independently of the composition of the melt. They also shows that the vaporization at constant temperature is incongruent, the gas being dominated by free Na and K cations and then, second in importance, small volatile species like O_2_, SiO, and SiO_2_. Vaporization leaves behind a melt that is enriched in Al and Si.

Compared to other available rock‐forming minerals, we find that both Na‐feldspars and K‐feldspars have critical temperatures located in the estimated range for silica, and below the critical temperature of MgSiO_3_. At the moment of a giant impact, materials can attain much higher temperatures, as observed in our Hugoniot equations of state, reaching a complete supercritical phase. Upon cooling of the disk, the MgSiO_3_ fluid would be the first to hit the liquid‐vapor dome if allowed to decompress, and start to separate into two phases, liquid and vapor. The Na‐feldspar, closely followed by the K‐feldspar, will remain in the supercritical state for a longer time. When these phases hit the liquid‐vapor dome, they would exhibit a strong chemical incongruent behavior and enrich the gas in alkalis.

Finally, our simulations suggest that impacts on a cold crust could melt the crust and lead to the formation of local magma lakes, like on the Moon. However, large and very large impacts on warm or even molten crust could have pushed the temperature in the first stages of the protolunar disk to extreme values, on the order of 20000–30000K. In this case previous giant impact simulations that consider hot or even molten initial states for the proto‐Earth might need to be revisited using these new Hugoniot equations of state.

## Supporting information



Supporting Information S1

## Data Availability

All the data sets referenced in the main text and in the supporting information are available in the Zenodo repository Kobsch and Caracas (2020).
