# Supplementary material for: The Critical Point and the Supercritical State of Alkali Feldspars: Implications for the Behavior of the Crust During Impacts
Source: J Geophys Res Planets. 2020 Sep 15;125(9):e2020JE006412. doi: 10.1029/2020JE006412 (PMC7583489; doi:10.1029/2020JE006412)
Supplement: Supplementary file 1 — Supporting Information S1 [file JGRE-125-e2020JE006412-s001.pdf]

Supporting Information for "The critical point and the supercritical state of alkali feldspars: implications for the behavior of the crust during impacts"

Anaïs Kobsch[1], Razvan Caracas[1,2]

[1]CNRS, École Normale Supérieure de Lyon, Laboratoire de Géologie de Lyon UMR 5276, 46 allée d'Italie, 69364 Lyon, France

[2]The Centre for Earth Evolution and Dynamics (CEED), University of Oslo, Postbox 1028 Blindern, N-0315 Oslo, Norway

Contents of this file

1. Figures S1 to S5
2. Tables S1 to S4

Additional Supporting Information (Files uploaded separately)

1. Captions for Data Sets S1 to S5

Introduction This supporting information provides every additional figures and tables which are mentioned in the article. It provides also the name and caption of every data set of thermodynamic parameters for both feldspars. All these data sets are available in the Zenodo repository "Thermodynamics data of Alkali Feldspars from FPMD simulations" (`http://doi.org/10.5281/zenodo.3860527`).

These data were computed in the scope of the three-years PhD thesis entitled "Behavior of feldspars during the Giant Impact", by Anaïs Kobsch. Many additional informa-

tion about the post-processing of data and results obtained are available in the thesis manuscript (published online on `tel.archives-ouvertes.fr` after the defense).

Data Set S1. Thermodynamic properties (pressure, temperature, internal energy, heat capacity) and thermoelastic coefficients (isobaric expansivity, isothermal compressibility, thermal pressure coefficient) computed from our ab-initio molecular dynamics simulations on $NaAlSi_3O_8$.

Arithmetic time averages of the pressure (P), temperature (T) and internal energy (E) are performed over the entire simulations. The standard deviation of the data to the mean is indicated by *stdev_X*, where X is P, T or E. The statistical error to the mean (*err_X*) is computed using the blocking method as described by Flyvbjerg and Petersen (1989). The sign '>' is indicated before the value of the statistical error when no convergence was reached during the estimation of this error. The heat capacity Cv is computed using fluctuations on both potential and kinetic energies (Allen & Tildesley, 1989) and its statistical error *stdev_Cv* is computed using the bootstrap method.

We computed the thermoelastic coefficients only for densities above $1.5\,\mathrm{g\,cm^{-3}}$. The thermal pressure coefficient ($TPC = \frac{\partial P}{\partial T}|_V$) is the slope of linear fit of P vs. T isochores. The isothermal compressibility ($\beta = -\frac{1}{\rho}\frac{\partial \rho}{\partial P}|_T$) is computed using central finite differences on our P vs. ρ isotherms. The isobaric expansivity ($\alpha = \frac{1}{\rho}\frac{\partial \rho}{\partial T}|_P$) is computed using the previously computed β and TPC.

Data are available in the file kobsch-ds01.txt

Data Set S2. Same as Data Set S1 for the simulations at low density using pseudopotentials which require a lower plane wave energy cutoff, set to 370 eV. Data are available in the file file kobsch-ds02.txt.

Data Set S3. Same as Data Set S1 for the simulations at high density, using hard pseudopotentials in order to reduce the overlap of electronic spheres, in particular for Na-

Na pairs. The energy cutoff for this set of pseudopotentials is 950 eV. Data are available in the file kobsch-ds03.txt.

Data Set S4. Same as Data Set S1 for $KAlSi_3O_8$. Data are available in the file kobsch-ds04.txt.

Data Set S5. Same as Data Set S1 for $KAlSi_3O_8$ and for the simulations at low density using pseudopotentials which require a lower plane wave energy cutoff, set to 370 eV. Data are available in the file kobsch-ds05.txt.

References

Allen, M. P., & Tildesley, D. J. (1989). *Computer Simulation of Liquids (Oxford Science Publications).* Clarendon Press.

Flyvbjerg, H., & Petersen, H. G. (1989). Error estimates on averages of correlated data. *The Journal of Chemical Physics*, *91*(1), 461–466.

Raymond, S. N., Schlichting, H. E., Hersant, F., & Selsis, F. (2013). Dynamical and collisional constraints on a stochastic late veneer on the terrestrial planets. *Icarus*, *226*(1), 671–681.

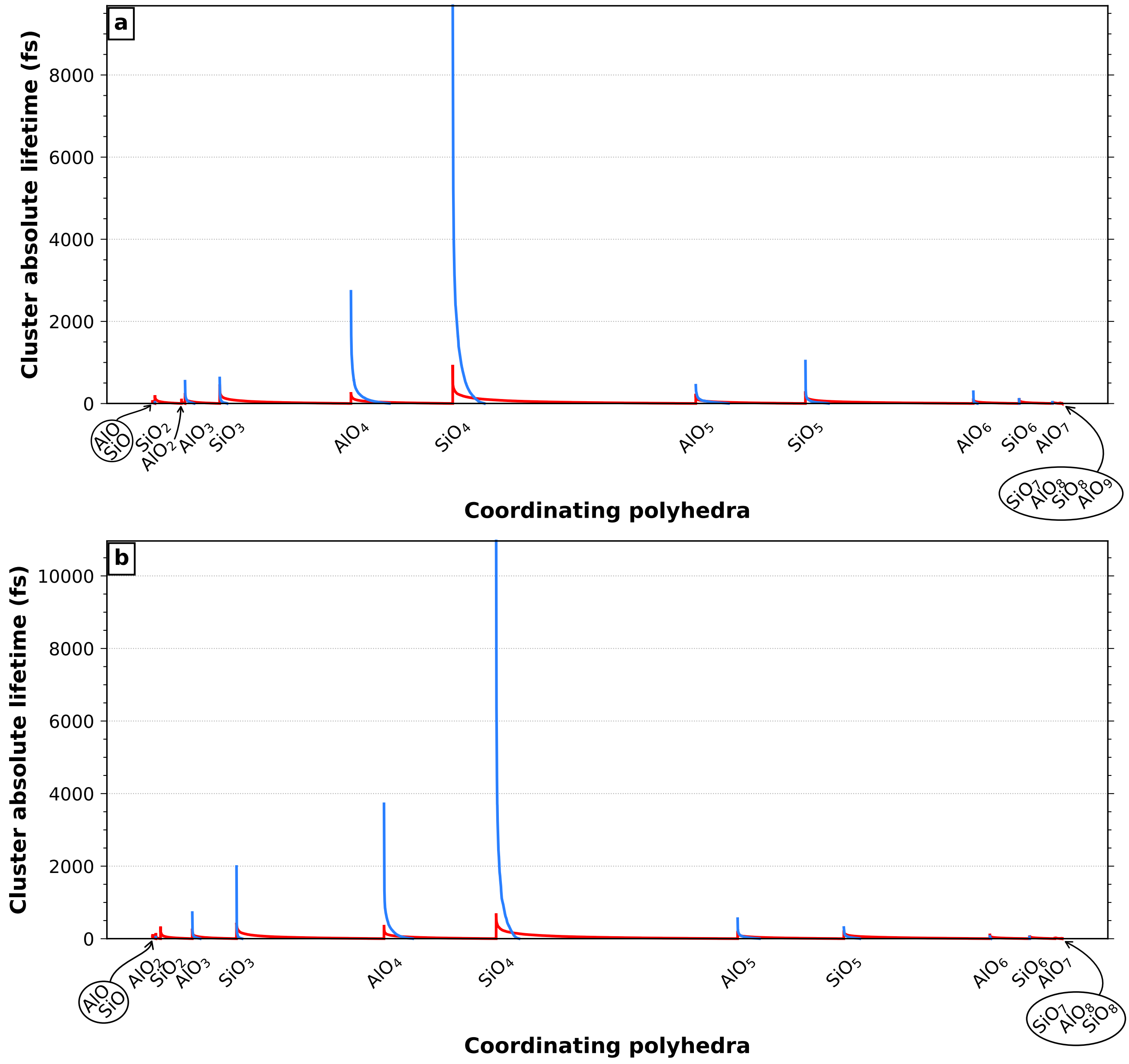

Figure S1. Lifetime of SiO_x and AlO_x coordinating polyhedra at about $2.2\,\mathrm{g\,cm^{-3}}$ below the critical temperature (blue - 3000 K) and above the critical temperature (red - 6000 K) for (a) Na- and (b) K-feldspar fluids. Due to the large number of individual species among each coordinating polyhedra, we display here the outline of the initial bar plot. The height of the each individual bar corresponds to the lifetime of each individual cluster.

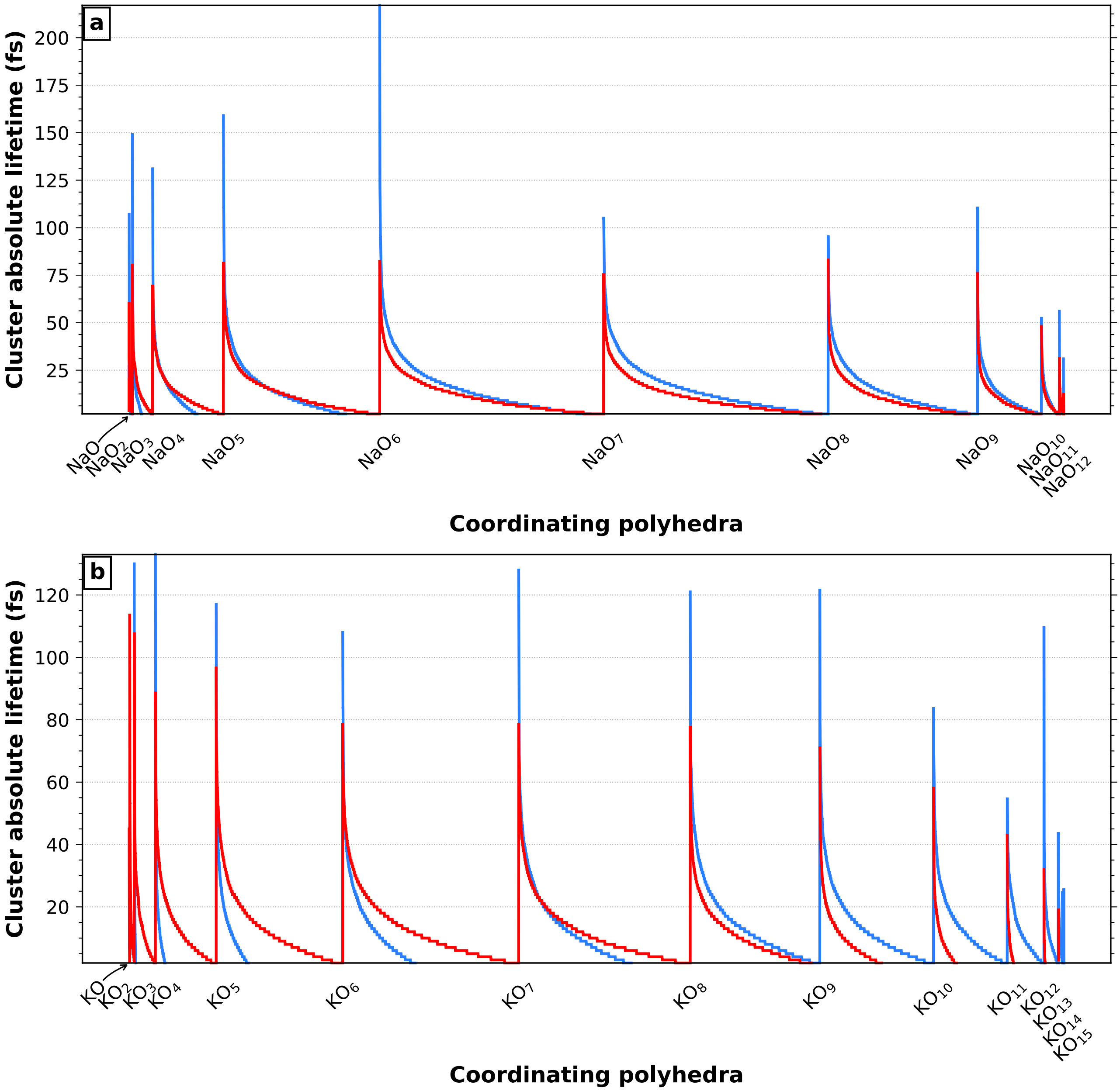

Figure S2. Same as Fig. S1 for NaO_x and KO_x coordinating polyhedra.

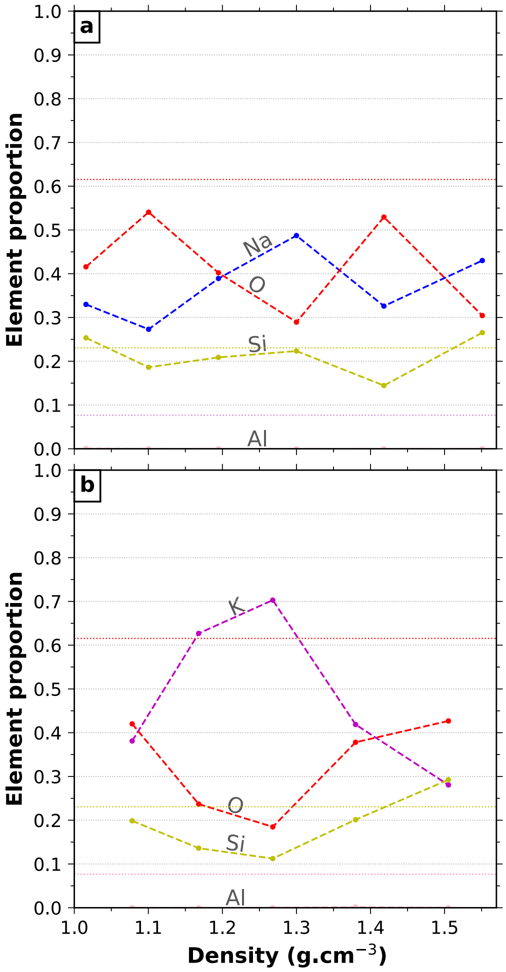

Figure S3. Proportion of each element in the group of small species (bottom species in figure 9, constituting the gas phase as a function of density at 4000 K for (a) Na- and (b) K-feldspar end-members. Color indicates the element type. The colored dotted horizontal lines indicate the congruent vaporization proportions: $R_{Na}=R_K=R_{Al}=7.7\,\%$, $R_O=61.5\,\%$ and $R_{Si}=3R_{Al}=23.1\,\%$. Volatilization is clearly incongruent since there is almost no Al in its composition.

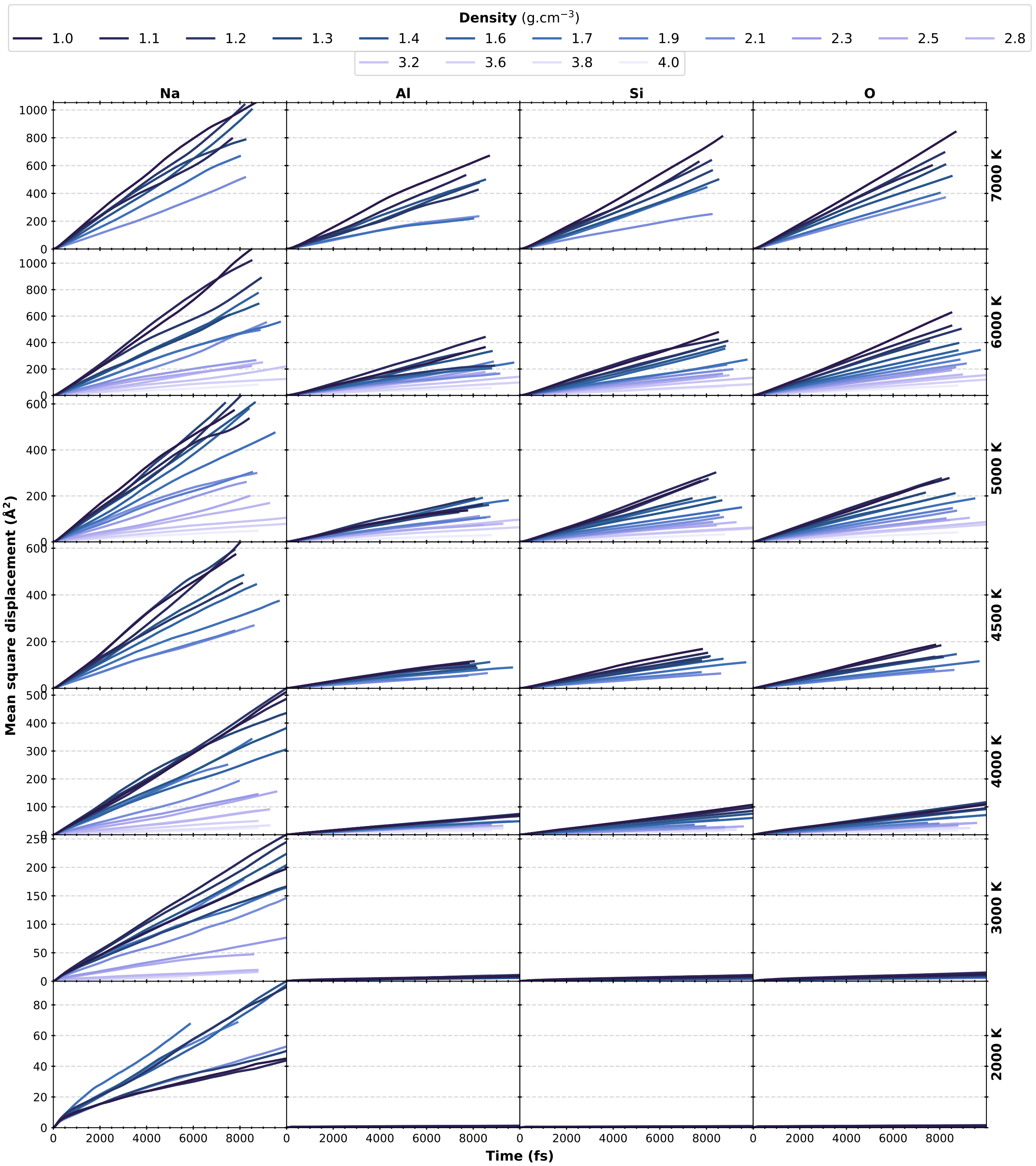

Figure S4. Mean square displacement of Na, Al, Si and O in $NaAlSi_3O_8$ at seven temperatures between 2000 K and 7000 K and up to 16 densities between $1.0\,g\,cm^{-3}$ and $4.0\,g\,cm^{-3}$.

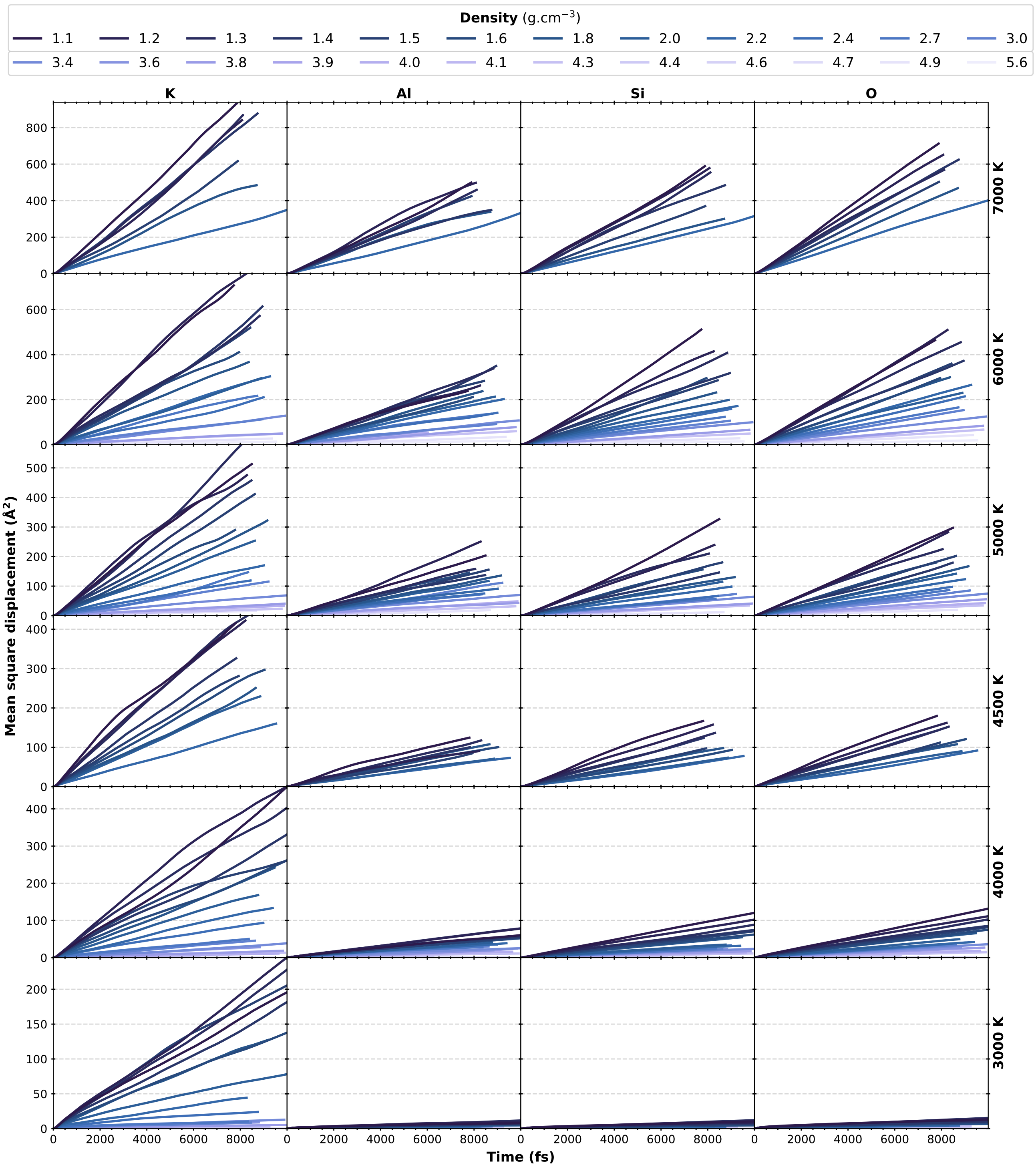

Figure S5. Mean square displacement of K, Al, Si and O in $KAlSi_3O_8$ at six temperatures between 3000 K and 7000 K and up to 24 densities between 1.1 g cm^{-3} and 5.6 g cm^{-3}.

Table S1. Temperature (T), density (ρ), pressure (P), energy (E) and particle velocity (Up) of the Hugoniot curves for the two alkali feldspars and the five initial state of the crust.

$NaAlSi_3O_8$					$KAlSi_3O_8$				
T (K)	ρ ($g\,cm^{-3}$)	P (GPa)	E ($MJ\,kg^{-1}$)	Up_{crust} ($km\,s^{-1}$)	T (K)	ρ ($g\,cm^{-3}$)	P (GPa)	E ($MJ\,kg^{-1}$)	Up_{crust} ($km\,s^{-1}$)
0	2.50	0	-36	0	0	2.50	0	-34	0
3000	4.61	80	-29	3.8	3000	4.61	80	-26	3.8
4000	4.73	97	-27	4.3	4000	4.72	95	-25	4.2
5000	4.86	116	-25	4.8	5000	4.87	115	-23	4.7
6000	4.97	136	-22	5.2	6000	4.95	130	-21	5.1
10000	5.32	213	-13	6.7	10000	5.23	191	-14	6.3
15000	5.60	297	-2.9	8.1	15000	5.50	260	-5.4	7.5
20000	5.81	373	6.6	9.2	20000	5.64	312	1.0	8.3
0	2.60	0	-36	0	0	2.60	0	-34	0
3000	4.70	86	-28	3.9	3000	4.71	87	-26	3.9
4000	4.83	105	-27	4.3	4000	4.83	104	-24	4.3
5000	4.97	126	-24	4.8	5000	4.96	123	-22	4.8
6000	5.10	148	-22	5.3	6000	5.06	141	-20	5.1
10000	5.47	231	-13	6.8	10000	5.37	207	-13	6.4
15000	5.76	321	-2.0	8.2	15000	5.65	281	-4.5	7.6
20000	5.95	397	7.1	9.3	20000	5.82	339	2.4	8.5
0	2.70	0	-36	0	0	2.70	0	-34	0
3000	4.79	94	-28	3.9	3000	4.82	95	-26	3.9
4000	4.94	115	-26	4.4	4000	4.93	111	-24	4.3
5000	5.09	138	-24	4.9	5000	5.06	133	-22	4.8
6000	5.23	162	-21	5.4	6000	5.17	152	-20	5.2
10000	5.62	251	-12	7.0	10000	5.51	225	-12	6.5
15000	5.92	345	-1.0	8.3	15000	5.77	298	-4.2	7.7
20000	6.11	423	7.9	9.4	20000	5.97	363	3.2	8.6
1932	2.585	4	-33	0	1932	2.585	6	-31	0
3000	4.01	43	-30	2.3	3000	3.92	41	-28	2.2
4000	4.30	66	-28	3.1	4000	4.25	63	-26	2.9
5000	4.51	88	-25	3.7	5000	4.45	82	-24	3.5
6000	4.68	110	-23	4.3	6000	4.63	102	-22	4.1
10000	5.17	195	-14	6.1	10000	5.04	170	-14	5.6
15000	5.50	283	-3.5	7.6	15000	5.37	243	-6.0	6.9
20000	5.76	366	6.5	8.8	20000	5.53	297	0.3	7.8
3000	2.26	2	-31	0	3000	2.26	2	-29	0
4000	3.56	30	-29	2.1	4000	3.56	31	-27	2.1
5000	3.86	48	-27	2.9	5000	3.87	48	-25	2.9
6000	4.09	68	-24	3.6	6000	4.08	64	-23	3.5
10000	4.59	136	-16	5.5	10000	4.53	122	-16	5.1
15000	4.94	211	-5.6	7.1	15000	4.82	178	-8.3	6.4
20000	5.16	278	3.6	8.3	20000	4.99	224	-2.1	7.3

Table S2. Temperature (T), density (ρ), pressure (P), energy (E) and particle velocity (Up) of the Hugoniot curves for the two alkali feldspars and the five initial velocities of the impactor.

	T (K)	ρ ($g\,cm^{-3}$)	P (GPa)	E ($MJ\,kg^{-1}$)	$Up_{impactor}$ ($km\,s^{-1}$)				
$NaAlSi_3O_8$	0	3.00	0	-36	8.3	11.5	12.9	15.2	18.1
	3000	5.13	124	-27	4.2	7.4	8.8	11.1	14.0
	4000	5.30	150	-25	3.7	6.9	8.3	10.6	13.5
	5000	5.44	175	-22	3.2	6.4	7.8	10.1	13.0
	6000	5.58	203	-20	2.7	5.9	7.3	9.6	12.5
	10000	6.00	306	-10	1.2	4.4	5.8	8.1	11.0
	15000	6.37	422	1.7	-0.3	2.9	4.3	6.6	9.5
	20000	6.62	518	12	-1.4	1.8	3.2	5.5	8.4
$KAlSi_3O_8$	0	3.00	0	-33	8.3	11.5	12.9	15.2	18.1
	3000	5.08	117	-25	4.3	7.5	8.9	11.2	14.1
	4000	5.22	139	-23	3.9	7.1	8.5	10.8	13.7
	5000	5.40	167	-21	3.3	6.5	7.9	10.2	13.1
	6000	5.55	196	-18	2.8	6.0	7.4	9.7	12.6
	10000	5.85	270	-11	1.7	4.9	6.3	8.6	11.5
	15000	6.08	345	-4.1	0.7	3.9	5.3	7.6	10.5
	20000	6.41	440	5.8	-0.5	2.7	4.1	6.4	9.3

Table S3. Shock states parameters for five different ground states and impactor velocities for the Na-feldspar. All the results with a temperature higher than 20 000 K are extrapolated from a linear fit of the Hugoniot data points. We choose velocities for the impactor of 12.9, 15.2 and 18.1 km s^{-1} for the impact with the Earth, and of 8.3, 11.5 and 15.2 km s^{-1} for the impact with the Moon. These values correspond respectively to the first, second, and third quartile of the 1487 impacts generated for the Earth in the work of Raymond et al. (2013) on planetary impacts during the late veneer. All their impactors had a density of 3.0 g cm^{-3}. Without information on their composition we consider them to be made of the same material as the crust.

$U_{impactor}$ ($km.s^{-1}$)	ρ_0 ($g.cm^{-3}$)	T_0 (K)	ρ ($g.cm^{-3}$)	T (K)	P (GPa)	U_p ($km.s^{-1}$)
8.3	2.26	3000	4.35	8029	102	4.65
8.3	2.585	1932	4.70	6200	114	4.39
8.3	2.5	0	4.80	4552	107	4.54
8.3	2.6	0	4.87	4278	111	4.46
8.3	2.7	0	4.94	3994	114	4.38
11.5	2.26	3000	4.78	12621	175	6.38
11.5	2.585	1932	5.16	9933	193	6.06
11.5	2.5	0	5.19	8522	185	6.21
11.5	2.6	0	5.28	8024	190	6.12
11.5	2.7	0	5.38	7507	196	6.02
12.9	2.26	3000	4.95	15179	213	7.13
12.9	2.585	1932	5.32	12253	234	6.79
12.9	2.5	0	5.36	10681	225	6.94
12.9	2.6	0	5.47	10000	231	6.84
12.9	2.7	0	5.56	9391	237	6.74
15.2	2.26	3000	5.20	20292	283	8.36
15.2	2.585	1932	5.59	16650	310	7.98
15.2	2.5	0	5.61	15089	299	8.14
15.2	2.6	0	5.72	14214	307	8.03
15.2	2.7	0	5.82	13389	315	7.92
18.1	2.26	3000	5.61	27502	385	9.92
18.1	2.585	1932	5.97	23142	420	9.48
18.1	2.5	0	5.92	22008	406	9.65
18.1	2.6	0	6.03	21087	417	9.52
18.1	2.7	0	6.13	20106	427	9.4

Table S4. Same as in Table S3 for the K-feldspar.

$U_{impactor}$ ($km.s^{-1}$)	ρ_0 ($g.cm^{-3}$)	T_0 (K)	ρ ($g.cm^{-3}$)	T (K)	P (GPa)	U_p ($km.s^{-1}$)
8.3	2.26	3000	4.38	8663	102	4.65
8.3	2.585	1932	4.71	6732	115	4.37
8.3	2.5	0	4.81	4631	107	4.54
8.3	2.6	0	4.88	4371	111	4.46
8.3	2.7	0	4.94	4141	114	4.38
11.5	2.26	3000	4.81	14804	176	6.38
11.5	2.585	1932	5.15	11665	194	6.05
11.5	2.5	0	5.20	9577	185	6.21
11.5	2.6	0	5.29	8987	190	6.12
11.5	2.7	0	5.38	8401	196	6.03
12.9	2.26	3000	4.95	18892	214	7.13
12.9	2.585	1932	5.34	14524	236	6.78
12.9	2.5	0	5.36	12477	225	6.95
12.9	2.6	0	5.46	11658	232	6.85
12.9	2.7	0	5.56	10902	238	6.75
15.2	2.26	3000	5.27	25714	284	8.36
15.2	2.585	1932	5.62	20968	312	7.97
15.2	2.5	0	5.60	18854	300	8.15
15.2	2.6	0	5.73	17312	308	8.04
15.2	2.7	0	5.82	16382	316	7.93
18.1	2.26	3000	5.74	35753	387	9.91
18.1	2.585	1932	6.06	29764	423	9.48
18.1	2.5	0	5.98	27825	408	9.66
18.1	2.6	0	6.11	25846	419	9.53
18.1	2.7	0	6.19	24698	429	9.41
